# Distinct glycosyltransferase expression governs organ-specific survival in pancreatic cancer metastasis

**DOI:** 10.1038/s41388-026-03896-5

**Published:** 2026-07-30

**Authors:** Venkatesh Varadharaj, Frank Leon, Nivedeta Krishna Kumar, Palanisamy Nallasamy, Pratima Raut, Annant Bir Kaur, Kirtana Arikath, Wyatt Petersen, Sanchita Rauth, Neelanjana Gayen, Zahraa Wajih Alsafwani, Poompozhil Mathivanan, Saravanakumar Marimuthu, Kavita Mallya, Madhu Bommideni, Jesse L. Cox, Geoffrey A. Talmon, Paul M. Grandgenett, Michael A. Hollingsworth, Dominick J. DiMaio, Jean L. Grem, Surinder K. Batra, Moorthy P. Ponnusamy

**Affiliations:** 1 Department of Biochemistry and Molecular Biology, University of Nebraska Medical Center, Omaha, NE USA; 2 Department of Cancer Research, University of Nebraska Medical Center, Omaha, NE USA; 3 Department of Pathology and Microbiology, University of Nebraska Medical Center, Omaha, NE USA; 4 Fred and Pamela Buffett Cancer Center, Eppley Institute for Research in Cancer and Allied Diseases, University of Nebraska Medical Center, Omaha, NE USA; 5 Department of Internal Medicine, University of Nebraska Medical Center, Omaha, NE USA

**Keywords:** Pancreatic cancer, Glycobiology, Prognostic markers, Glycobiology

## Abstract

Pancreatic ductal adenocarcinoma (PDAC) is among the most lethal cancers, with over 83% of patients presenting metastases to vital organs such as the liver, lungs, and peritoneum. Although glycosylation has been established as a critical factor in PDAC development, little is known about the molecular underpinnings of organ-specific metastasis. In this study, we investigated the role of glycosyltransferases (GTs) in mediating PDAC metastatic organotropism. Through an unbiased transcriptomic screen, we identified distinct GT expression patterns in liver- and lung-tropic PDAC cells. Notably, GCNT3 and B3GNT3 were selectively upregulated in liver and lung-tropic cells, respectively. These mutually exclusive expression patterns were validated in human metastatic PDAC specimens, where GCNT3 was enriched in liver metastatic lesions and B3GNT3 in lung metastases. Proteomic profiling revealed that differential expression of these GTs is associated with epithelial-mesenchymal transition and glycoproteome remodeling in metastatic cells. Functional studies using in vitro assays, ex vivo organotypic cultures, and in vivo metastasis models demonstrated that loss of GCNT3 or B3GNT3 impairs the proliferation and survival of PDAC cells in their respective metastatic organ environments. Together, these findings uncover a glycosylation-based mechanism that facilitates organ-specific survival of metastatic PDAC cells and highlight glycosyltransferases as potential therapeutic targets for disrupting metastatic adaptation.

**Figure:**
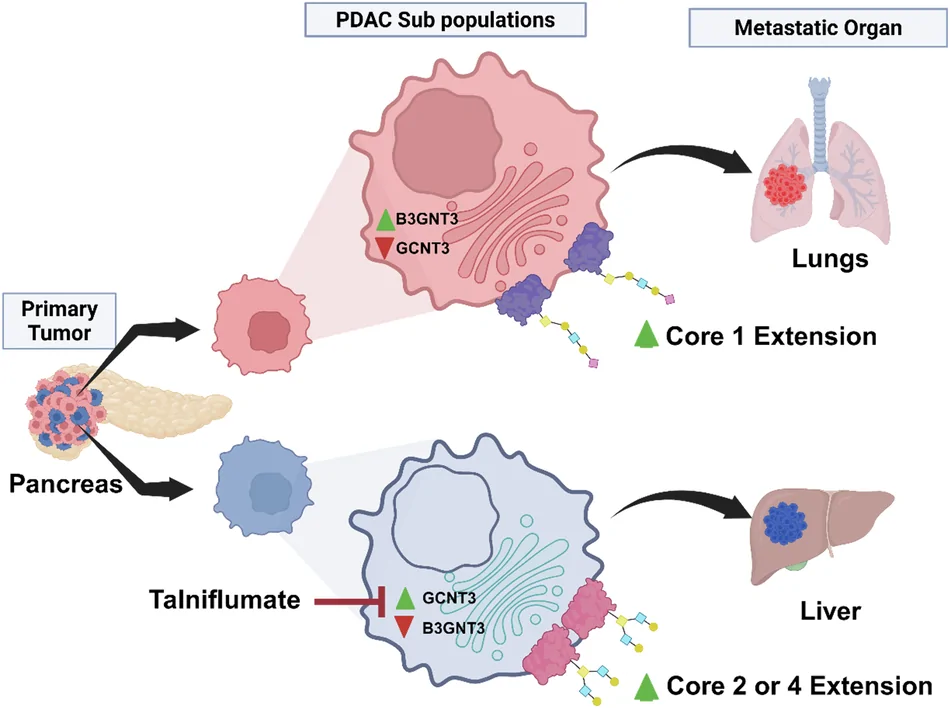

## Introduction

PDAC remains one of the most lethal malignancies globally and is projected to become the second leading cause of cancer-related fatalities in the United States by 2030 [1, 2]. The five-year survival rate of patients with PDAC is less than 13%, mainly because it tends to metastasize at an early stage of tumor development, and due to the lack of early detection [3]. By the time PDAC is diagnosed, ~60% of patients already have metastatic cancer at a stage that cannot be surgically removed [4].

A major challenge lies in deciphering the metastatic cascade, particularly the mechanisms by which tumor cells establish colonies in distant organs, and identifying the factors that drive organ-specificity [5]. PDAC metastasis primarily occurs in vital organs such as the liver and lungs. Many PDAC patients relapse with liver metastasis within two years of surgery [6–8]. Even though patients with lung metastases have better survival, the overall prognosis remains poor for many patients, highlighting that lung metastasis does not inherently guarantee better outcomes [9, 10]. Diagnostic imaging and tissue biopsy are the cornerstones of PDAC clinical management, and surgical resection is considered a curative intervention. Despite these significant clinical measures, the long-term outcomes for patients with PDAC remain poor [11]. Therefore, probing a unique molecular mechanism that specifically targets the metastatic perspective of cancer will increase the survival prospects of PDAC patients.

Notably, the accumulation of immature O-glycans, such as Tn and sialyl-Tn (STn) antigens, has been closely linked to these oncogenic features [12–14]. Proteomic heterogeneity in PDAC contributes to organ-specific metastasis, with alterations in cell surface glycosylation further amplifying this diversity [15]. Emerging studies highlight O-glycosylation as a critical modulator of protein function, yet the contribution of aberrant O-glycan structures in PDAC remains underexplored [16–19]. GTs involved in O-glycan biosynthesis influence these protein activities by perturbing extracellular signaling and modulating downstream cellular responses [20, 21].

In this study, we demonstrated that distinct GT expression profiles are associated with lung- and liver-specific metastasis in patients with PDAC. To uncover these patterns, we began with an unbiased analysis of GT expression changes in organ-enriched PDAC metastatic cells derived from the lung and liver. Two key GTs, glucosaminyl-N-acetyl transferase 3 (GCNT3), and UDP-GlcNAc, beta-Gal beta-1,3-N-acetylglucosaminyl-transferase 3 (B3GNT3), were identified as key regulators preferentially expressed in liver- and lung-tropic cells, respectively. Functional studies revealed that loss of either GT impaired migratory and clonogenic potential. Proteomic analyses further identified glycoprotein targets altered by these GTs, suggesting mechanistic roles in metastatic colonization. Our findings highlight a glycosylation-dependent regulatory axis that shapes organ-specific metastasis, potentially by modulating EMT pathways, and offer new insights into PDAC progression.

## Methods

### Patients and specimens

Human primary tumor samples, as well as matched liver (*n* = 8) and lung metastases (*n* = 9) from individuals previously diagnosed with PDAC, were obtained through the UNMC Rapid Autopsy Program, following IRB protocol 091–01. To preserve tissue integrity, organs were collected within three hours of death and either flash-frozen in liquid nitrogen or fixed immediately in formalin. Formalin-fixed tissues were embedded in paraffin, sectioned at 4 µm thickness, and mounted on charged slides. Additionally, healthy human liver and lung tissues (*n* = 1) from the RAP were used to generate biomatrix scaffolds for 3D ex vivo metastasis models.

### Animals

Male and female athymic nude mice aged 6–8 weeks were used in the study. Animal experiments were conducted under the protocol approved by the Institutional Animal Care and Use Committee of the University of Nebraska Medical Center, Omaha, Nebraska. All animal experiments were conducted following the National Institutes of Health guidelines for the care and use of laboratory animals.

### Cell lines and culture conditions

Cell lines BEAS-2B (ATCC® CRL-9609™), MRC-5 (ATCC® CCL-171™), HUVEC (ATCC® PCS-100-010™), and HEK-293T cells were obtained from the American Type Culture Collection and authenticated by short tandem repeat (STR) DNA profiling and were routinely tested for mycoplasma contamination monthly. Human immortalized hepatocytes (Fa2N-4, Cat# IFH15), along with MFE support and plating media, were obtained from XenoTech LLC (Lenexa, KS). The S2-VP10 pancreatic cancer cell line was generously provided by Dr. Ashok K. Saluja (University of Miami, USA), while SUIT-2 and L3.6pL cell lines were kindly provided by Dr. Michel Ouellette (UNMC). The LX-2 hepatic stellate cell line was a generous gift from Dr. Scott L. Friedman, Dean for Therapeutic Discovery and Chief of the Division of Liver Diseases at the Icahn School of Medicine at Mount Sinai, New York, USA. Pancreatic cancer cell lines and metastatic cell line models were maintained in Dulbecco’s modified Eagle’s medium (DMEM; 4.5 mg/mL glucose) supplemented with 10% fetal bovine serum (FBS) and 1% penicillin‒streptomycin (100 units/mL penicillin and 100 μg/mL streptomycin). The BEAS-2B cell line was cultured using medium purchased from Lonza (Cat# CC-3170). The MRC5 fibroblast cell line was maintained in Eagle’s Minimum Essential Medium (ATCC Cat# 30–2003), supplemented with 10% fetal bovine serum (FBS) and antibiotics (100 U/mL penicillin and 100 μg/mL streptomycin). Human umbilical vein endothelial cells (HUVECs) were grown in Vascular Cell Basal Medium (ATCC® PCS-100-030™), supplemented with components from the Endothelial Cell Growth Kit-BBE (ATCC® PCS-100-040™). The LX-2 hepatic stellate cell line was cultured in Dulbecco’s Modified Eagle Medium (DMEM; 4.5 mg/mL glucose), supplemented with 2% FBS and antibiotics (100 U/mL penicillin and 100 μg/mL streptomycin). The cells were incubated at 37 °C in a humidified atmosphere with 5% CO₂. For the second metastatic model, we harvested a pancreatic tumor, metastatic liver spots (Liver met 2), and metastatic lung spots (Lung met 2). Briefly, we chopped and minced the tissues and dissolved them in RPMI containing Liberase (1 mg/ml) for 30 min in a water bath at 37 °C. Following tissue solubilization, cells were passed through a single-cell strainer and cultured for 10 passages. Differential trypsinization was performed at each passage to specifically select ductal PC cells by avoiding other cell contaminations, such as fibroblasts. These cell lines were used at less than passage 15. These primary cell lines were grown adherently in RPMI medium supplemented with 10% fetal bovine serum and antibiotics.

### Orthotopic tumor implantation

PDAC metastatic model cell lines, with or without GCNT3 or B3GNT3 KO, were prepared at a concentration of 0.5 × 10⁶ cells per mouse. The cells were suspended in 50 µL of sterile PBS and orthotopically injected into the pancreas tail of randomized 6- to 8-week-old athymic nude mice under isoflurane anesthesia. Each experimental group consisted of 5 mice, and all animals were sacrificed at a fixed experimental endpoint (45 days post-implantation). Samples size of *N* = 5 for each condition, based on our previous studies and no statistical methods were used to determine sample size. The primary tumors were harvested, weighed, and assessed for metastatic burden in the lung and liver.

### Lung- and liver-specific ex vivo metastatic models

To model organ-specific metastatic colonization, we developed ex vivo lung and liver metastatic niche systems using decellularized tissue scaffolds reconstituted with tissue-resident stromal components. For the lung model, human lung epithelial cells (BEAS-2B), fibroblasts (MRC5), and endothelial cells (HUVECs) were sequentially seeded onto decellularized lung ECM scaffolds and incubated for 48 h. Similarly, a liver model was established by co-seeding primary human hepatocytes (Fa2N4), hepatic stellate cells (LX-2), and HUVECs onto decellularized liver scaffolds. After reconstitution of the respective stromal niches, spheroids derived from equal numbers of either lung-tropic or liver-tropic metastatic cells were overlaid onto the scaffolds and cultured under 3D conditions for 5 days. At the endpoint, the scaffolds were fixed in 4% PFA, embedded in paraffin, sectioned, and processed for IHC and IF. The number of spheroids that survived in the lung or liver metastatic niche served as a readout for tumor cell survival within metastatic microenvironments.

### MTT cytotoxicity assay

To assess cell viability, 3000 cells per well were seeded in 96-well plates and allowed to adhere for 24 h before treatment. The cells were then exposed to a range of talniflumate concentration for 48 h, with 6 replicates per concentration. Following treatment, MTT reagent (5 mg/mL) was added to each well, and the samples were incubated for 4 h at 37 °C. The medium was subsequently removed, and 200 µL of DMSO was added to solubilize the formazan crystals. The absorbance was measured via a microplate reader (BMG Labtech), and the relative cell viability was calculated on the basis of the absorbance values.

### Droplet digital PCR (ddPCR)

EvaGreen-based ddPCR was performed on the Bio-Rad QX200 system, which comprises of a droplet generator, a thermal cycler, and a droplet reader. cDNA was synthesized from total RNA as described for qPCR and quantified via a NanoDrop spectrophotometer. Reactions were assembled via ddPCR EvaGreen Supermix (Bio-Rad), with 10 μL of supermix, 1–2 μL of cDNA template (10–50 ng), target-specific primers (optimized at 50 nM and 150 nM for singleplex and duplex formats), and nuclease-free water to a final volume of 22 μL. Droplets were generated by loading 22 μL of the reaction mixture into DG8 cartridges along with droplet generation oil. The emulsified droplets were transferred to a 96-well PCR plate and subjected to thermal cycling: initial denaturation at 95 °C for 10 min; 40 cycles of 94 °C for 30 s and 60 °C for 1 min; and signal stabilization at 98 °C for 10 min. A ramp rate of 2 °C/sec was used throughout. The plates were then read via a QX200 droplet reader, and each sample had three technical replicates. The data were analyzed via QuantaSoft software to calculate the absolute DNA concentration (copies/μL) for each gene. For normalization and comparative analysis, the housekeeping gene ActB was used in both singleplex and duplex formats. Primer titrations allowed clear separation of droplet clusters in 1D amplitude plots, enabling accurate quantification of GCNT3 and B3GNT3 across all PDAC cell lines. Duplex reactions with different primer concentrations were used to simultaneously detect target genes and ActB, maintaining high specificity and minimal cross-channel interference.

### RT‒PCR array

The expression levels of target genes were analyzed via a specific RT² Profiler™ PCR Array (QIAGEN, Hilden, Germany). Complementary DNA (cDNA) was synthesized as mentioned in qPCR. For the RT‒PCR array, the cDNA template was mixed with a ready-to-use RT² SYBR® Green qPCR Mastermix (QIAGEN). The reaction mixture was prepared by combining 1350 µL of Mastermix with 102 µL of cDNA template, followed by the addition of nuclease-free water to a final volume of 2700 µL. This reaction mixture was then aliquoted into each well of a 96-well RT² Profiler™ PCR Array plate, with each well receiving 25 µL of the mixture. The PCR array plate was then subjected to real-time PCR amplification with the following cycling conditions: 1 cycle of 10 min at 95 °C for enzyme activation, followed by 40 cycles of 15 s at 95 °C (denaturation) and 1 min at 60 °C (annealing/extension). Data acquisition was performed at the end of each annealing/extension step, and threshold cycle (Ct) values were automatically calculated via the instrument’s software. The Ct values were normalized to the average of the housekeeping genes included in the array, and relative gene expression was calculated via the ΔΔCt method.

## Results

### Differential expression of GT in lung and liver metastatic PDAC cells

**Fig. 1:**
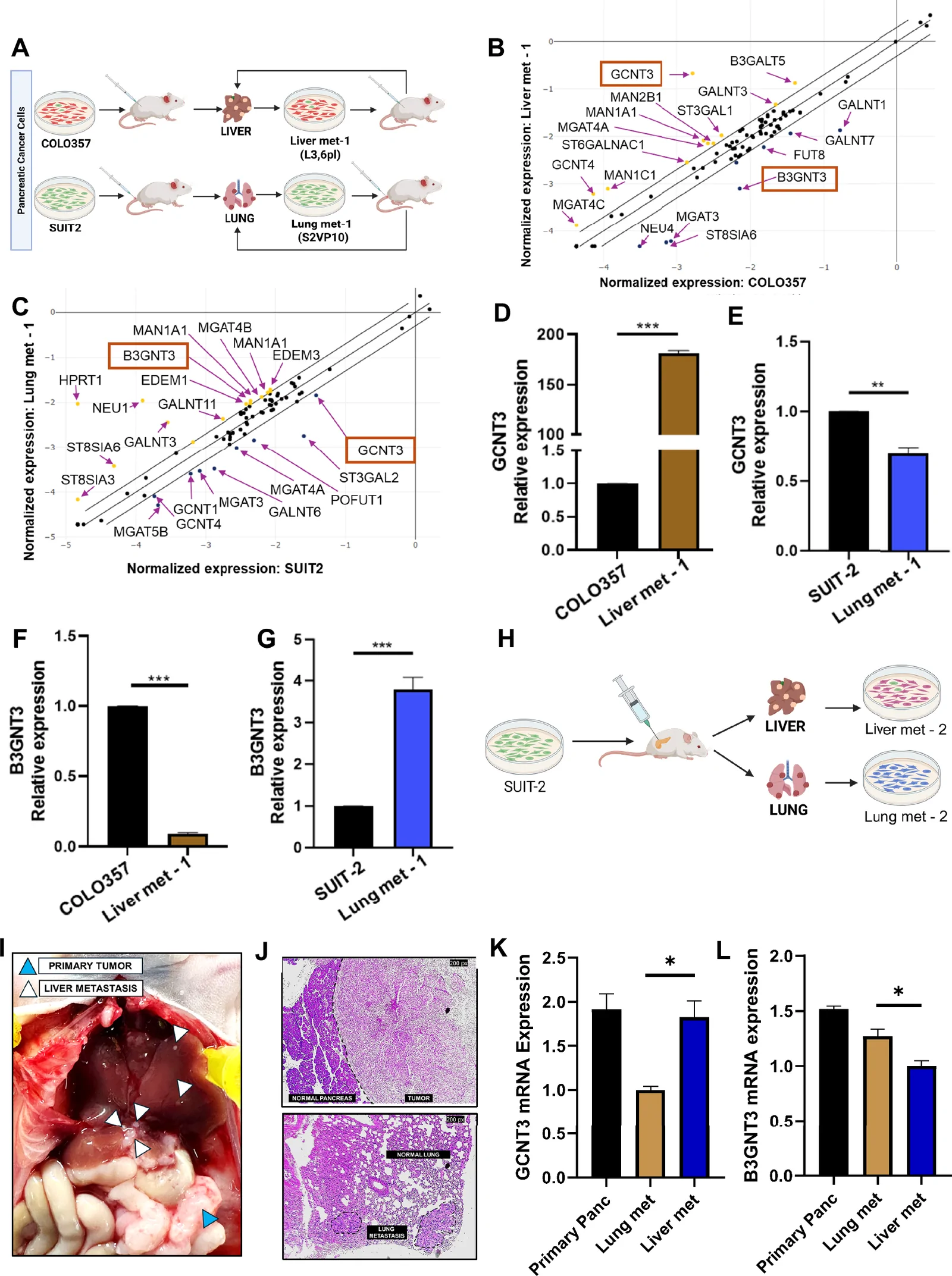
Differential expression of GT in lung/liver metastatic PDAC cells. **A** Schematic representation of the 1st PDAC metastatic model developed through serial in vivo selection of liver-tropic (L3.6pL) and lung-tropic (S2VP10) metastatic subclones from the Colo357 and SUIT-2 parental cell lines, respectively. **B**, **C** RT² Profiler PCR array profiling identifies glycosyltransferases that are differentially expressed between parental and organ-tropic metastatic derivatives. Liver-tropic vs. Colo357 parental (**B**); lung-tropic vs. SUIT-2 parental (**C**). **D**, **E** qPCR validation demonstrated significantly increased GCNT3 expression in liver-tropic cells (**D**) and decreased GCNT3 expression in lung-tropic cells (**E**), which is consistent with the array findings. **F**, **G** qPCR validation demonstrated significantly decreased B3GNT3 expression in liver-tropic cells (**F**) and elevated B3GNT3 expression in lung-tropic cells (**G**), which was consistent with the array findings. **H** Schematic representation of the 2nd metastatic model showing the derivation of matched primary tumors, liver metastases, and lung metastases after orthotopic implantation of SUIT-2 cells into the pancreas of immunocompromised mice. **I** Gross morphology of a representative mouse pancreas and associated liver metastases from the second model, confirming successful liver colonization. **J** Representative H&E staining of primary pancreatic tumors and matched lung metastatic lesions in the second model, validating the lung metastatic phenotype. **K**, **L** The second metastatic model confirmed the organ-specific enrichment of GCNT3 (**K**) in liver metastases and B3GNT3 (**L**) in lung metastases, validating the reproducibility of the GT expression patterns across the models. The data are presented as the means ± SEMs. Statistical significance was determined by a two-tailed Student’s *t* test unless otherwise indicated. *P < 0.05; **P < 0.01; ***P < 0.001; NS not significant.

Recent studies have identified key GTs associated with the cancer phenotype and elucidated their mechanistic roles in driving PDAC progression [22, 23]. To investigate whether GT expression patterns are specifically linked to metastasis, we utilized two different organ-tropic PDAC metastatic models. It includes liver-enriched metastatic cells (L3.6pL – Liver met 1) and lung-enriched metastatic cells (S2VP10 – Lung met 1), derived through iterative cycles of in vivo selection involving orthotopic implantation and tail vein injection of COLO357 (celiac lymph node metastatic cell line) and SUIT-2 (liver metastatic cell line), respectively (Fig. 1A) [24, 25]. Although both enriched metastatic cells colonize other secondary sites, S2VP10 cells preferentially and robustly form lung metastases, while L3.6pL cells predominantly colonize the liver, indicating a dominant, reproducible organ-specific colonization pattern. We conducted an unbiased transcriptional analysis of human GTs and observed more than twofold upregulation of GTs in the lung and liver metastatic cell lines compared with their corresponding parental PDAC cells (Fig. 1B, C). Among these GTs, GCNT3 showed the most pronounced expression, specific to liver metastatic cells (Fig. 1B, D, E), whereas B3GNT3 was more highly expressed in lung metastatic cells, as confirmed by q-PCR analysis (Fig. 1C, F, G). To independently validate the link between metastatic organotropism and GT expression, a secondary model was generated via the orthotopic implantation of SUIT-2 cells, followed by the isolation of cancer cells from the primary tumor, liver metastasis (Liver met 2) (Fig. 1H, I), and lung metastasis (Lung met 2) (Fig. 1H, J). We then validated the GCNT3 and B3GNT3 expression pattern in the second metastatic model (Fig. 1K, L). Other GTs did not show significant organ-specific changes in their expression pattern either in the RT² Profiler PCR Array or upon qPCR validation (Supplementary Fig. 1A and Table 1A). These findings demonstrate organ-specific GT expression profiles in the lung and liver metastatic subclones of PDAC.

**Table 1:** List of primers used and their sequences (A) and the shRNA sequence used for the knockdown studies (B).

A		
Target	Direction	Sequence (5’ → 3’)
B4GALT5	Forward	CCAAGAAAGCAGTCCGATGA
	Reverse	TCTCTTGCTGTGTAGACCCT
B3GNT3	Forward	CTCACAGACGGGATGCTAAG
	Reverse	GACGGAGCTTATCTGCCATT
EDEM1	Forward	GCTACTGCTATGGTTTCAGGA
	Reverse	ATTTCCATCCCCCTTTCACC
EDEM3	Forward	ATCCGGGAATATGAGGAGGT
	Reverse	ATTGCTGCTCGATCTTTTGC
FUT8	Forward	TGAAGACTTGAGGCGAATGG
	Reverse	TAGCTGGCCCCTGATCAATA
GALNT1	Forward	AATGGCCCAGTTACAATGCT
	Reverse	ATACTCCCAGAGTTGGTTGC
GALNT11	Forward	GACAATGGCCCTTCAGCTAT
	Reverse	AAAGGCACCCATAACAGAAA
GALNT14	Forward	TCATCACCTTCCACAACGAG
	Reverse	GGGGTGCGGTTTAATACACT
GALNT3	Forward	GAATGGCTCACCTAAAGCGA
	Reverse	GCACCAAGCTTCCAGAACTT
GALNT6	Forward	ACAACTGCACTGTCACAACT
	Reverse	CGTCAGGTCAGGAACAAACA
GALNT7	Forward	AGAGTTGTGGAGGTTTGGTG
	Reverse	ATGGTAATGCCTGCGATTCA
GCNT1	Forward	GCATAGAAGACTGCCCTTCA
	Reverse	AAAAGTCTCCTTCGCAGCAA
GCNT3	Forward	CTCAGTGCCGTGGAAATACT
	Reverse	GACCATCTCTGCATTGCTCT
GCNT4	Forward	AGACGACTCTTTCCGCAAAA
	Reverse	AAAGGCGAGGTACTTAGGGA
GLB1	Forward	CCTCAATGGAGTCCACGATC
	Reverse	TTGTTTCGCTCAAGGACTCC
HPRT1	Forward	GTGAAAAGGACCCCACGAAG
	Reverse	TCCTACAACAAACTTTGTCTGGA
MAN1A1	Forward	CCAGAATAACTTCTCCCACTCA
	Reverse	GGAACAAAGGGCTCTTGCTA
MAN1A2	Forward	CACCTTGCAGGAGTTCAAGA
	Reverse	TGAATGCTGCTCCACTGTAG
MAN1C1	Forward	TGCTTGGCAGAGCTATAAGC
	Reverse	TGTTACCCTCGTAGCCATCT
MAN2B1	Forward	GGAAACTGAACCAGACGGAG
	Reverse	CGTGATGTAAATCCGGGTGT
MGAT3	Forward	TCCAACTTCACGGCTTATGG
	Reverse	TGTACTCGAAGGTGCCATTG
MGAT4A	Forward	GAATCTCACAGCGCTCTTCT
	Reverse	CTCCTACACGCTTGAACTGT
NEU1	Forward	GATGGCTCAGTCGTCATCAA
	Reverse	ATAGCTGCGGAGGACAATTC
NEU4	Forward	CCGAGAGTGTTTTGGCAAGA
	Reverse	GTGGTCATCGCTGTAGAAGG
POFUT1	Forward	TAAACCGTACCTTGGCTGTC
	Reverse	GACACATGGAGGTTGGTGAA
ST3GAL1	Forward	TTTGATTGAGCCTCCACCTC
	Reverse	GCAGGAACTCCCTCCTAAGA
ST3GAL2	Forward	GTAACCACTTTGGCCTCTGT
	Reverse	ATGTATCGGAGCCTGATGGA
ST6GALNAC1	Forward	AGGTGAGTGCTTATGGCTTC
	Reverse	AGCCGCTTCCATGATGTATC
ST8SIA3	Forward	CTTCACCACTCCCAAGTACG
	Reverse	GCAAATTGTGACCGGAATCC
ST8SIA6	Forward	GCTGTGATGCTGTTCAAAAC
	Reverse	GCTTTCCACCTCGTAACTCA

B	
shRNA target	Sequence
GCNT3	GACCCAAAGGTAGATGATAAT
B3GNT3	AGGTTTGATGAGTGAATATTC

### Selective overexpression of GCNT3 and B3GNT3 defines organ-specific glycan signatures in metastatic PDAC models

**Fig. 2:**
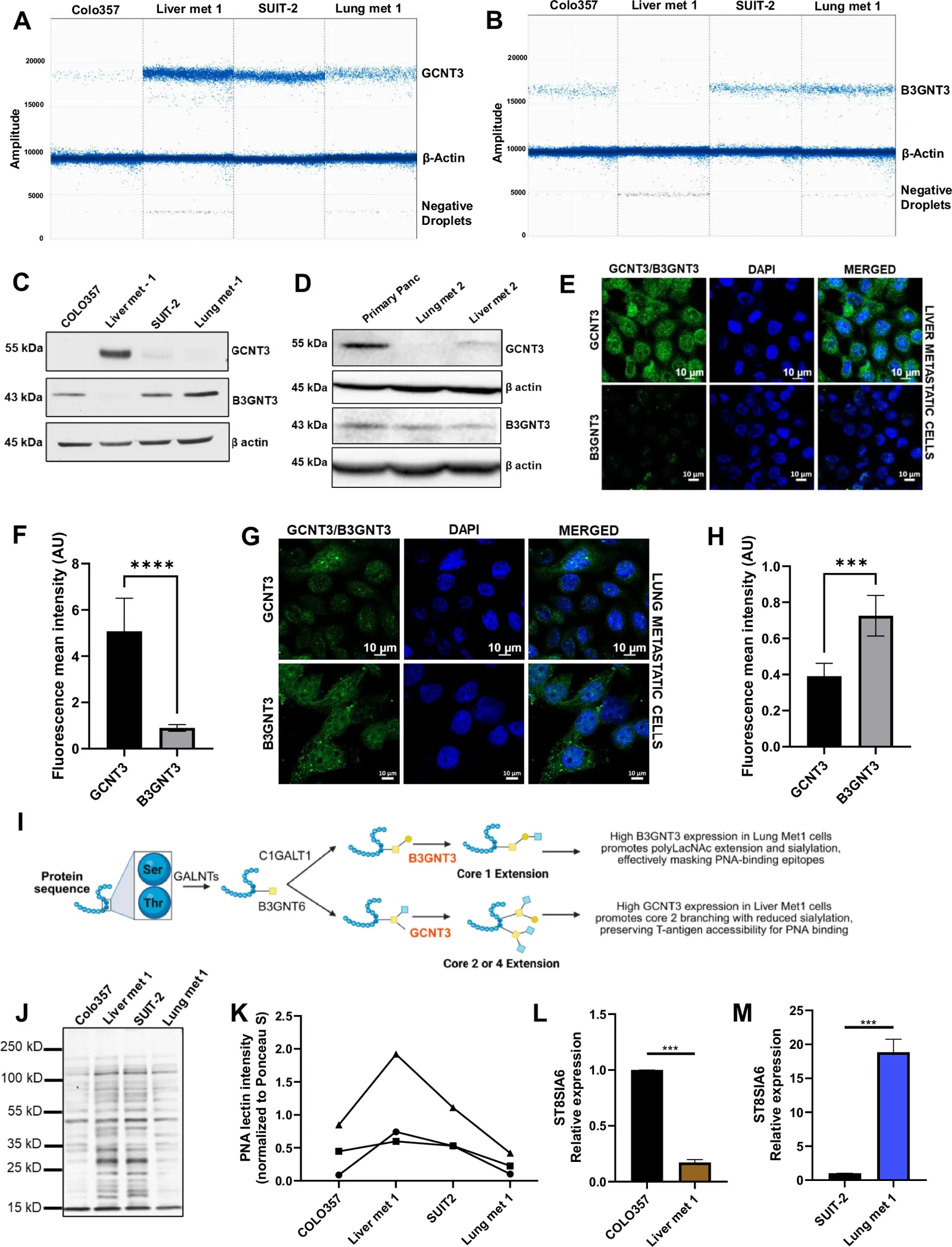
Selective overexpression of GCNT3 and B3GNT3 defines organ-specific glycan signatures in metastatic PDAC models. **A**, **B** Droplet digital PCR (ddPCR) analyses of GCNT3 (**A**) and B3GNT3 (**B**) transcript levels in liver- and lung-tropic PDAC cells compared with those in the Colo357 and SUIT-2 parental cell lines, normalized to β-actin. **C** Western blot analysis confirmed elevated GCNT3 expression in liver-tropic cells and B3GNT3 expression in lung-tropic cells compared with the Colo357 and SUIT-2 parental cell lines. **D** Second metastatic model validates organ-specific GT expression patterns at the protein level. GCNT3 is more highly expressed in liver-tropic cells, and B3GNT3 is more highly expressed in lung-tropic cells. **E**–**H** Immunofluorescence imaging further demonstrated site-selective expression of GCNT3 in liver-tropic cells (**E**, **F**) and B3GNT3 in lung-tropic cells (**G**, **H**). **I** Schematic overview of the roles of the O-glycan pathway for GCNT3 and B3GNT3 in shaping glycan structures and how it influences PNA binding. **J** PNA lectin blot showing increased PNA binding in liver-tropic cells compared with lung-tropic cells, consistent with preservation of T antigen in liver metastasis and reduced accessibility of PNA epitopes in lung-tropic cells due to increased sialylation. **K** Quantification of PNA lectin intensity at three molecular weight ranges (~25, 50, and 75 kDa), demonstrating significantly higher PNA reactivity in liver-tropic cells relative to lung-tropic cells. RT‒qPCR revealed that the differential expression of the sialyltransferase ST8SIA6 was correlated with the observed sialylation patterns in the liver (**L**) and lung metastatic subtypes (**M**). The data are presented as the means ± SEMs. *P < 0.05; **P < 0.01; ***P < 0.001; NS not significant.

The differential expression of GTs plays a critical role in regulating organ-specific metastasis by influencing cancer cell interactions with distinct metastatic microenvironments [26–29]. To validate the overexpression of GCNT3 and B3GNT3 in liver- and lung-specific metastatic cells and to determine the absolute transcript levels, droplet digital PCR (ddPCR) was performed via an EvaGreen-based detection strategy. The ddPCR results confirmed a clear separation between positive and negative droplets, indicating robust amplification and high assay specificity. Quantitative analysis revealed substantial enrichment of GCNT3 in Liver met1 cells (Fig. 2A and Supplementary Fig. 1B) and B3GNT3 in Lung met1 cells (Fig. 2B and Supplementary Fig. 1C), corroborating the qPCR findings. This expression pattern was also observed in the second metastasis model, in which the liver met 2 and lung met 2 cells were compared (Supplementary Fig. 1D–G). Protein-level assessment by western blot analysis further validated these transcriptional trends (Fig. 2C). GCNT3 protein expression was significantly elevated in liver met1 cells and B3GNT3 in lung met1 cells compared with their respective primary PDAC controls, a pattern confirmed in the secondary metastatic model (Fig. 2D) and further validated by immunofluorescence analysis (Fig. 2E–H). To confirm that GT expression functionally alters protein glycan structures, we employed PNA lectin binding assays to assess changes in sialylation patterns. Although liver met1 cells have high GCNT3 expression, they preserve T antigen accessibility, resulting in stronger PNA binding. In contrast, lung met1 cells presented elevated B3GNT3 expression, leading to increased sialylation, effectively masking PNA-recognizable T-antigen epitopes (Fig. 2I–K and Supplementary Fig. 1H). These findings were further validated by RT‒qPCR (Fig. 2L, M), which revealed consistent changes in the expression of the sialyltransferase ST8SIA6 across both metastatic cell lines, and the findings were validated by ddPCR analysis (Supplementary Fig. 1I, J) along with KPC (Kras, Trp53, Pdx1-Cre mouse model) lung and liver metastasis samples (Supplementary Fig. 1K). Despite these site-specific molecular differences, no significant differences were observed in overall survival or disease-free survival between the high and low expression groups of ST8SIA6 (Supplementary Fig. 1L, M). Collectively, these data support the notion that organ-specific metastatic PDAC cells adopt distinct glycan signatures, with GCNT3 predominating in liver-tropic and B3GNT3 in lung-tropic metastatic phenotypes. This reciprocal expression pattern validates a potential mechanistic role for these GTs in driving site-specific colonization and adaptation during PDAC metastasis.

### Clinical correlation of organ-specific GT expression in human pancreatic cancer samples and metastatic lesions

**Fig. 3:**
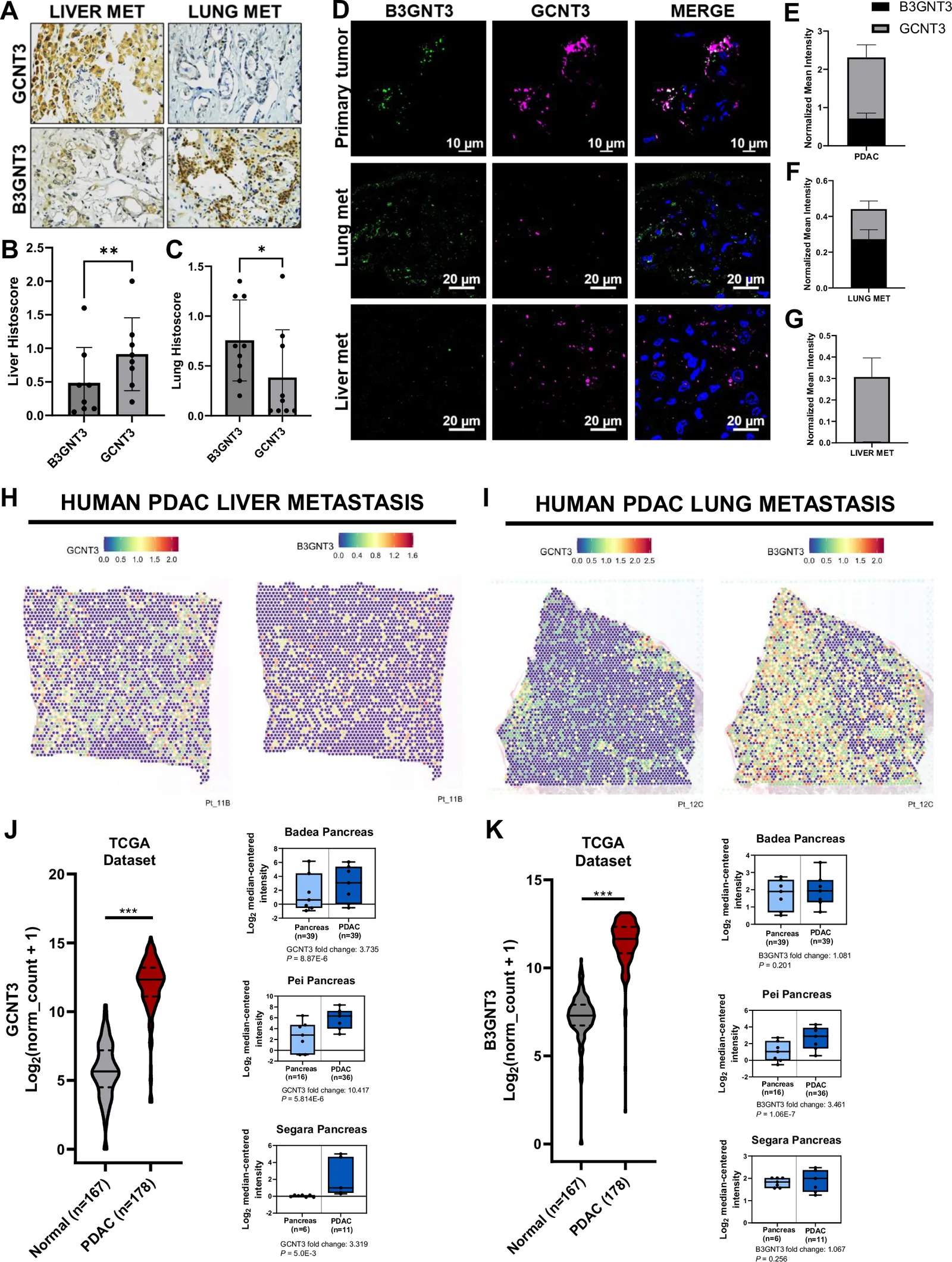
Clinical correlation of organ-specific GT expressions in human pancreatic cancer samples and metastatic lesions. **A** Immunohistochemical (IHC) analysis of human PDAC rapid autopsy samples revealed increased GCNT3 expression in liver metastases and elevated B3GNT3 expression in lung metastases. **B**, **C** Quantification of IHC staining confirming significant organ-specific enrichment of each GT in human PDAC liver (**B**) and lung (**C**) metastatic tissue sections. **D**–**G** Immunofluorescence imaging validated the differential expression of GCNT3 and B3GNT3 in human PDAC primary (**E**), lung (**F**) and liver (**G**) metastatic tissue sections. **H, I** Spatial transcriptomics (GSE274557) demonstrated distinct intratumoral enrichment of GCNT3 and B3GNT3 expression within liver (**H**) and lung (**I**) metastases, respectively. **J**, **K** Transcriptomic data from the TCGA and GTEx datasets showing elevated GCNT3 (**J**) and B3GNT3 (**K**) expression in PDAC tumors relative to normal pancreas. The data are presented as the means ± SEMs. *P < 0.05; **P < 0.01; ***P < 0.001; NS not significant.

To assess the clinical relevance of GCNT3 and B3GNT3 in organ-specific metastasis, we evaluated their expression in human PDAC lung (*n* = 9) and liver metastatic tissues (*n* = 8). Immunohistochemistry revealed elevated expression of GCNT3 in liver metastases (Fig. 3A, B) and increased B3GNT3 expression in lung metastases tissue samples (Fig. 3A, C). Immunofluorescence of metastatic tissues validated this expression pattern, and primary pancreatic tumor tissues showed that both subpopulations are present and express within the same tumor microenvironment (Fig. 3D–G). Furthermore, spatial transcriptomic analysis of GSE274557 demonstrated that GCNT3 and B3GNT3 expressions were analyzed within the liver or lung metastatic samples, revealing gene-specific spatial expression differences across patients (Fig. 3H, I) along with their primary tumor samples (Supplementary Fig. 2A). Differential expressions of GCNT3 and B3GNT3 were observed across patient primary tumor samples (Supplementary Fig. 2B–F), with corresponding clinical information on the metastasis (Supplementary Fig. 2G). Primary tumor spatial expression data were not available for patient 12 due to limited tumor tissue representation on the analyzed slide. Transcriptomic data from GTEx and TCGA revealed significantly higher expression of both genes in PDAC tumors than in normal pancreatic tissues (Fig. 3J, K), consistent across independent datasets. Notably, high GCNT3, B3GNT3, and both expression correlated with poorer overall survival (Supplementary Fig. 3A–C) and disease-free survival (Supplementary Fig. 3D–F), highlighting their prognostic potential. Furthermore, GCNT3 and B3GNT3 expression levels were positively correlated with ABSOLUTE purity scores (ρ = 0.24 and ρ = 0.65, respectively), indicating their predominant expression in tumor cells based on gene expression and methylation data [30] (Supplementary Fig. 3G, H). Among the glycogenes, both genes ranked among those with the highest fold change in PDAC (Supplementary Fig. 3I). To examine associations between GCNT3 and B3GNT3 expression and PDAC molecular subtypes, we analyzed the GSE71729 dataset stratified into classical and basal-like tumors. Subtype analysis showed that GCNT3 expression was significantly higher in classical PDAC tumors, whereas B3GNT3 did not display a subtype-specific difference (Supplementary Fig. 3J, K). Consistent with these findings, western blot analysis in our metastatic PDAC model revealed higher E-cadherin expression in liver-tropic metastatic cells and increased ZEB1 expression in lung-tropic cells (Supplementary Fig. 3L), supporting an association of GCNT3 with a classical epithelial phenotype and lung-tropic cells with basal-like features. Together, these findings support the tumor-intrinsic and site-specific roles of GCNT3 and B3GNT3 in PDAC metastasis and their potential as biomarkers and therapeutic targets.

### B3GNT3 loss impairs tumorigenic and metastatic traits in lung-tropic PDAC cells

**Fig. 4:**
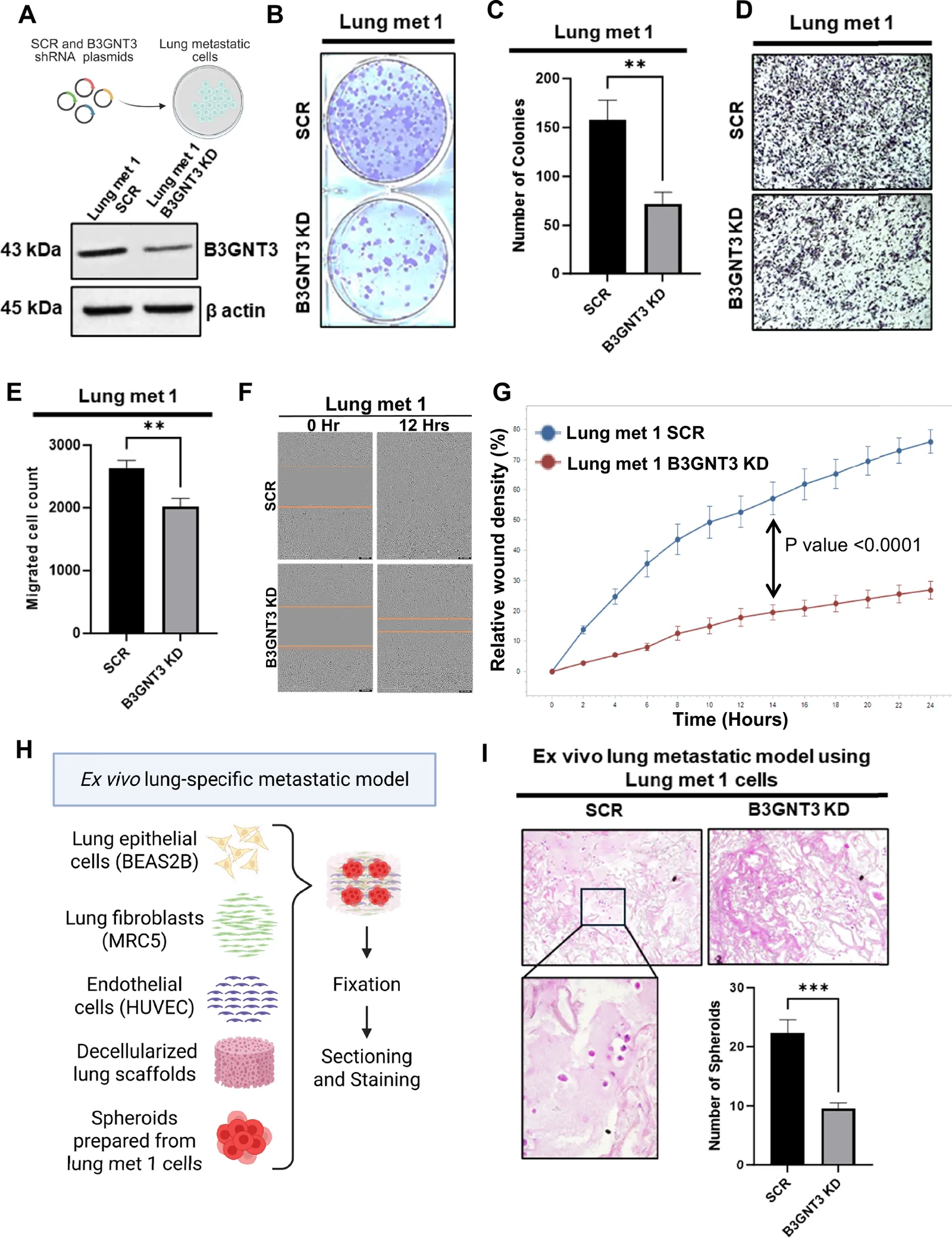
B3GNT3 loss impairs tumorigenic and metastatic traits in lung-tropic PDAC cells. **A** Western blot analysis confirmed efficient shRNA-mediated knockdown of B3GNT3 expression in lung-tropic PDAC cells (first metastatic model). **B**, **C** Clonogenic assays revealed a significant reduction in the colony-forming potential of B3GNT3 KD clones compared with that of scrambled control (SCR) clones (**B**), indicating impaired proliferative capacity and quantification (**C**). **D**, **E** Transwell migration assays revealed a marked decrease in migratory ability following B3GNT3 KD (**D**), supporting its role in promoting cell motility and quantification (**E**). **F**, **G** Wound-healing assays revealed delayed wound closure in B3GNT3-KD clones relative to SCR controls (**F**), further confirming the reduced migratory capacity and quantification (**G**). **H** Schematic illustration of the ex vivo lung metastasis model, which involves the incorporation of decellularized lung scaffolds cocultured with lung niche cells (BEAS2B cells, MRC5 cells, HUVECs) to mimic the lung microenvironment and evaluate organ-specific survival. **I** Quantification of 3D spheroid survival within the ex vivo lung model revealed a significant reduction in the number and viability of spheroids formed by B3GNT3 KO cells compared with those formed by control cells, indicating compromised lung-specific metastatic adaptation. The data are presented as the means ± SEMs. *P < 0.05; **P < 0.01; ***P < 0.001; NS not significant.

To investigate the functional role of B3GNT3 in lung-tropic PDAC cells, we employed shRNA-mediated knockdown (KD) (Fig. 4A) in the first metastatic model. B3GNT3 silencing led to a significant reduction in colony-forming ability, as shown by clonogenic assays (Fig. 4B, C), indicating impaired long-term proliferative capacity, which is a critical feature of metastatic colonization. Transwell migration assays revealed a marked decrease in cell motility upon B3GNT3 depletion (Fig. 4D, E), and wound-healing assays further confirmed delayed migration and reduced wound closure in KD cells (Fig. 4F, G). In our second metastatic model, we validated these findings via CRISPR/Cas9-mediated B3GNT3 KO (Supplementary Fig. 4A). Compared with the first model, B3GNT3 KO did not affect clonogenic potential (Supplementary Fig. 4B, C) but elicited a pronounced reduction in cell migration (Supplementary Fig. 4D, E) and impaired wound-healing capacity (Supplementary Fig. 4F), further reinforcing its role in promoting a prometastatic phenotype. To evaluate the role of B3GNT3 in a more physiologically relevant setting, we utilized an ex vivo lung metastasis-mimicking model consisting of 3D spheroids cocultured on decellularized lung scaffolds (Fig. 4H). In this model, compared with control spheroids, B3GNT3-deficient spheroids presented a significantly reduced number of spheroids, suggesting compromised survival within the lung-specific microenvironment (Fig. 4I). These results were further supported by an independent replication in the second model (Supplementary Fig. 4G). To further evaluate cellular plasticity and organ-specific adaptation, we performed reciprocal culture experiments in which liver- and lung-tropic metastatic PDAC cells were grown in liver- and lung-derived microenvironments. Liver-tropic metastatic cells displayed significantly enhanced survival in the liver microenvironment but showed markedly reduced survival when cultured in the lung microenvironment or when treated with O-glycan inhibitor BAG (benzyl-α-N-acetylgalactosamine). Conversely, lung-tropic metastatic cells survived only within the lung microenvironment and failed to persist in the liver microenvironment or with inhibitor, exhibiting near-complete loss of viable spheroids under these conditions. These reciprocal experiments provide internal controls and demonstrate a clear organ-specific survival advantage for metastatic cells within their corresponding microenvironments (Supplementary Fig. 4H–J). Collectively, these findings demonstrate that B3GNT3 promotes both the dissemination and lung-specific survival of PDAC cells, highlighting its functional importance in metastatic outgrowth within the lung microenvironment.

### Talniflumate treatment inhibits GCNT3 and reduces tumorigenesis and liver-tropic metastatic potential

**Fig. 5:**
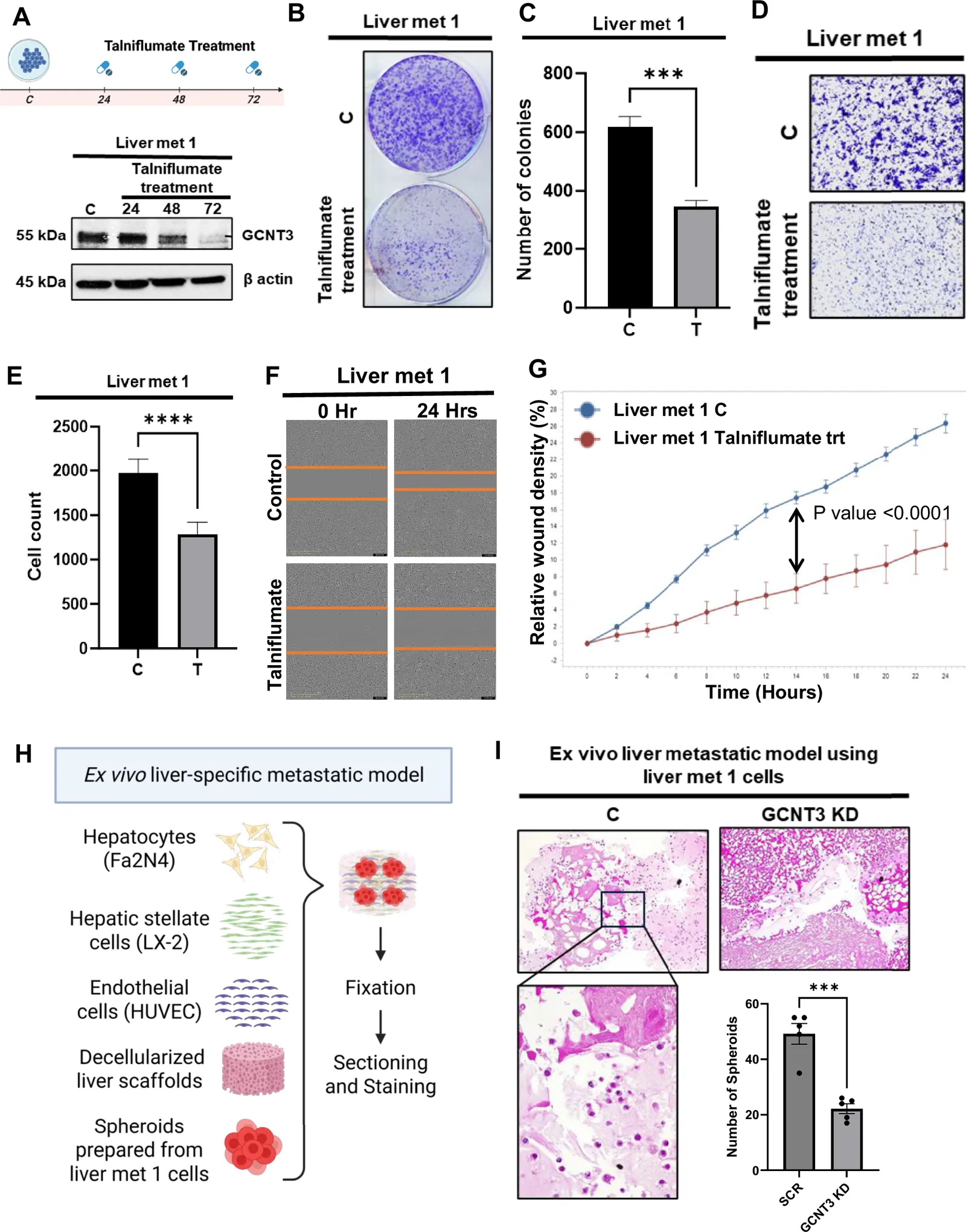
Talniflumate treatment inhibits GCNT3 and reduces tumorigenesis and liver-tropic metastatic potential. **A** Western blot analysis confirmed the time-dependent reduction in GCNT3 expression in liver-tropic PDAC cells following pharmacological inhibition with talniflumate at its IC50, demonstrating effective target engagement. **B, C** Clonogenic assays revealed that talniflumate-treated liver-tropic PDAC cells exhibited significantly reduced colony-forming capacity compared with untreated controls (**B**), indicating impaired long-term proliferative potential and quantification (**C**). **D, E** Transwell migration assays revealed a substantial decrease in the migratory ability of liver-tropic PDAC cells following GCNT3 inhibition with talniflumate (**D**), supporting a role for GCNT3 in promoting motility and quantification (**E**). **F, G** Wound-healing assays revealed delayed wound closure in talniflumate-treated cells relative to control cells (**F**), further confirming the reduction in migratory potential upon pharmacological targeting of GCNT3 and quantification (**G**). **H** Schematic representation of the ex vivo liver-specific metastasis model, which employs decellularized human liver scaffolds cocultured with liver stromal cells to mimic the native liver metastatic niche and assess organ-specific tumor cell survival. **I** Quantification of 3D spheroid survival within the ex vivo liver model revealed that, compared with control cells, GCNT3 KO cells display significantly reduced viability and survival, underscoring the essential role of GCNT3 in supporting PDAC colonization and adaptation within the liver microenvironment. The data are presented as the means ± SEMs. *P < 0.05; **P < 0.01; ***P < 0.001; NS not significant.

To investigate the role of GCNT3 in liver-tropic PDAC cells, we employed both talniflumate-based pharmacological inhibition [31] and CRISPR Cas9-mediated gene KO strategies. Talniflumate, used at its IC50 concentration (Supplementary Fig. 5A), selectively targets GCNT3 and leads to a time-dependent reduction in its expression (Fig. 5A). Compared with those of control cells, functional assessment via colony formation assays revealed a marked decrease in the clonogenic potential of talniflumate-treated cells (Fig. 5B, C). To assess cell motility, transwell migration assays revealed significantly reduced migration in GCNT3-inhibited cells (Fig. 5D, E), and wound healing assays revealed delayed closure, indicating impaired migration (Fig. 5F, G). To complement these findings, we generated a GCNT3 KO liver metastatic cell line via CRISPR-Cas9 (Supplementary Fig. 5B). Consistent with the pharmacological inhibition data, GCNT3 KO cells also presented significantly reduced colony formation capacity (Supplementary Fig. 5C, D). Transwell migration assays also revealed reduced migration in the KO cells (Supplementary Fig. 5E, F), and wound-healing assays revealed a lower relative wound density in the KO cells than in the control cells (Supplementary Fig. 5G). To evaluate the functional impact of GCNT3 in a liver-specific metastatic context, we employed an ex vivo liver metastasis-mimicking model in which 3D spheroid cultures were co-seeded onto decellularized liver scaffolds (Fig. 5H). In this physiologically relevant model, GCNT3 KO spheroids exhibited significantly reduced survival, forming fewer colonies than control liver-tropic spheroids (Fig. 5I). Collectively, these results demonstrate that GCNT3 promotes both the proliferation and organ-specific survival of PDAC cells within the liver metastatic niche, highlighting its functional importance in metastatic colonization and its potential as a therapeutic target.

### B3GNT3 and GCNT3 loss impairs the in vivo organ-specific survival of metastatic PDAC cells

**Fig. 6:**
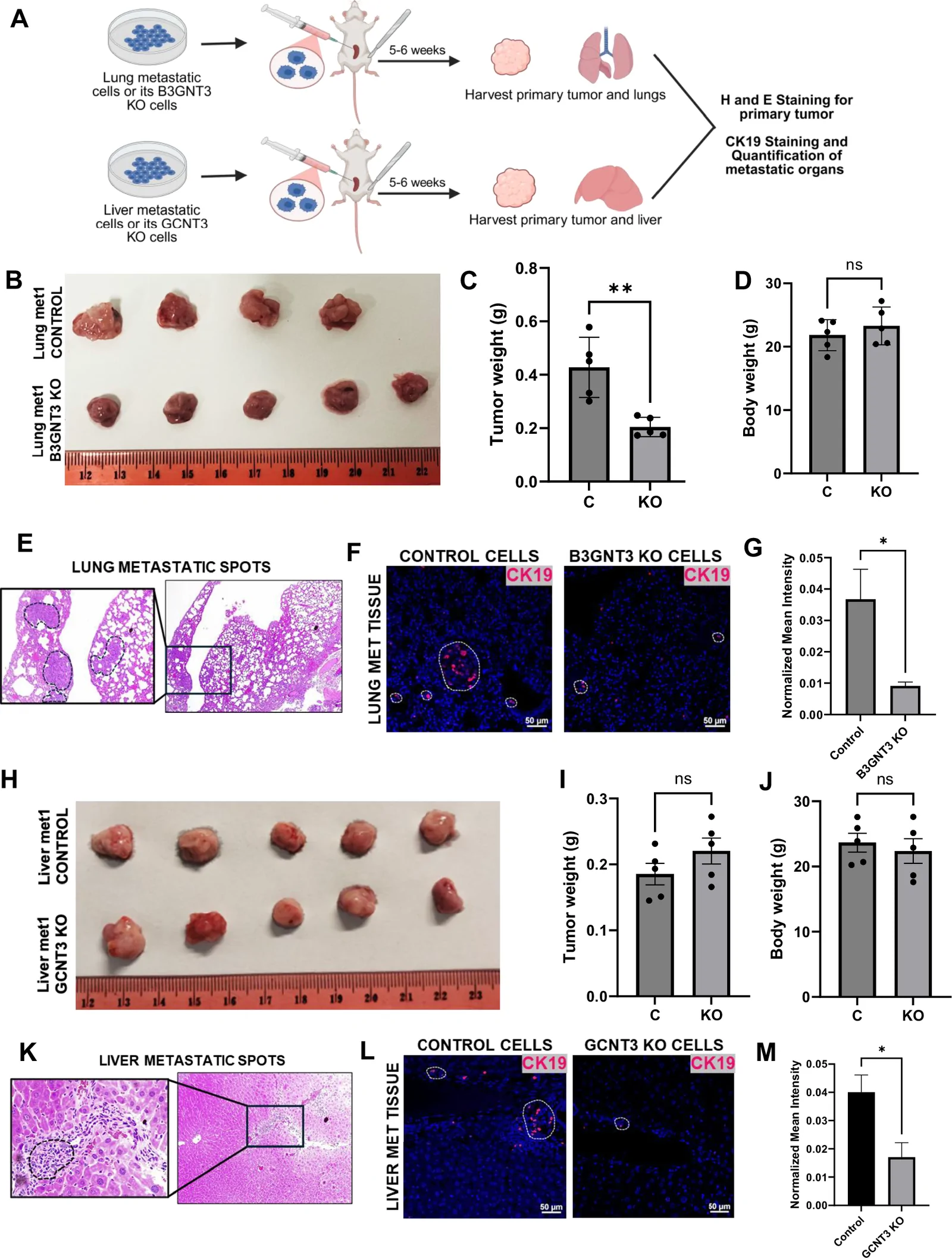
B3GNT3 and GCNT3 loss impairs the in vivo organ-specific survival of metastatic PDAC cells. **A** Schematic overview of the orthotopic implantation strategy: CRISPR/Cas9-engineered B3GNT3 KO lung-tropic and GCNT3 KO liver-tropic PDAC cells, along with corresponding control cells, were orthotopically injected into the pancreata of immunodeficient mice to assess their in vivo metastatic colonization capacity. **B**, **C** Orthotopic implantation experiments demonstrated significantly reduced primary tumor weight in B3GNT3 KO lung-tropic PDAC models (**B**), confirming the role of B3GNT3 in promoting in vivo tumor growth and metastatic fitness and changes in tumor weight (**C**). Note: One control animal developed ascites and was sacrificed before the experimental endpoint. Tumor tissue was recovered and included in tumor weight quantification, but no solid tumor image was available for display, resulting in four control tumors being shown in figure (**B**). **D** No significant differences in body weight were observed across treatment groups in the lung-tropic cohort, indicating that B3GNT3 KO-associated tumor reduction is not attributable to systemic toxicity. **E** Lung-tropic PDAC cells exhibit significantly increased numbers of metastatic lesions in the lung, as quantified by histological analysis of harvested lungs. **F**, **G** Immunofluorescence staining for CK19, an epithelial tumor marker, confirmed a markedly diminished burden of CK19-positive metastatic lesions in the lungs of mice implanted with B3GNT3-KO cells compared with those of control mice (**F**), validating the critical role of B3GNT3 in facilitating lung-specific metastatic colonization and quantification (**G**). **H**, **I** In the liver-tropic cohort, the primary tumor weight remained unchanged between the GCNT3-KO and control groups (**H**), suggesting that GCNT3 loss by CRISPR KO preferentially impaired metastatic potential rather than primary tumor growth in this model, as determined by quantification (**I**). **J** Body weight of mice in the liver-tropic cohort is not significantly affected by GCNT3 KO, further supporting the safety and specificity of targeting GCNT3 for liver metastasis inhibition. **K** Liver-tropic PDAC cells display pronounced formation of metastatic lesions within the liver, as observed by histological assessment of liver tissues. **L**, **M** CK19 staining further confirmed a significant reduction in the number and size of CK19-positive liver metastatic foci in mice implanted with GCNT3-KO cells compared with those in control mice (**L**), highlighting the functional requirement of GCNT3 for liver-specific metastatic adaptation and quantification (**M**).

To determine the functional roles of GTs B3GNT3 and GCNT3 in organotropic metastasis in PDAC, we used CRISPR-Cas9-mediated KO models in which each gene is targeted in PDAC cell lines. Isogenic control and KO cells were orthotopically implanted into the pancreata of immunodeficient mice to mimic physiological tumor progression and metastatic dissemination (Fig. 6A). In the lung-tropic model, compared with control tumors, B3GNT3-deficient tumors presented a reduction in primary tumor weight (Fig. 6B, C). However, this difference did not translate into systemic effects, as body weights remained unchanged (Fig. 6D). Notably, B3GNT3 loss profoundly impaired lung metastasis. While control mice developed multiple macroscopic metastatic lesions in the lungs (Fig. 6E), B3GNT3 KO tumors failed to produce visible metastases. CK19 immunofluorescence staining of lung sections confirmed this observation, revealing significantly fewer epithelial tumor cells in the lungs of B3GNT3 KO-bearing mice (Fig. 6F, G), underscoring the essential role of B3GNT3 in supporting pulmonary colonization. In parallel, we assessed the effect of GCNT3 KO in a liver-tropic PDAC model. While GCNT3 KO had no significant effect on primary tumor weight or body mass (Fig. 6H–J), the mice with control cells presented few hepatic metastatic burdens in liver tissues (Fig. 6K) but not in the mice with GCNT3 KO cells. This finding was further confirmed by CK19 staining, which revealed markedly fewer tumor cells in the liver parenchyma of mice injected with GCNT3-deficient cells (Fig. 6L, M), supporting the specific role for GCNT3 in hepatic colonization. Consistent with their organ-enriched phenotype, liver-tropic metastatic cells showed no detectable lung colonization (Supplementary Fig. 5H), whereas lung-tropic cells exhibited only minimal liver involvement (micro-metastatic foci) (Supplementary Fig. 5I). Taken together, these data provide compelling evidence that B3GNT3 and GCNT3 are not merely markers but also active mediators of site-specific metastatic adaptation in patients with PDAC. Their loss compromises the ability of cancer cells to establish and expand in distant organs, underscoring the functional requirement for these GTs in supporting metastatic fitness within the lung and liver microenvironments, respectively.

### Proteomic profiling revealed glycoprotein-driven changes in cellular pathways and EMT signatures in organ-tropic PDAC cells

**Fig. 7:**
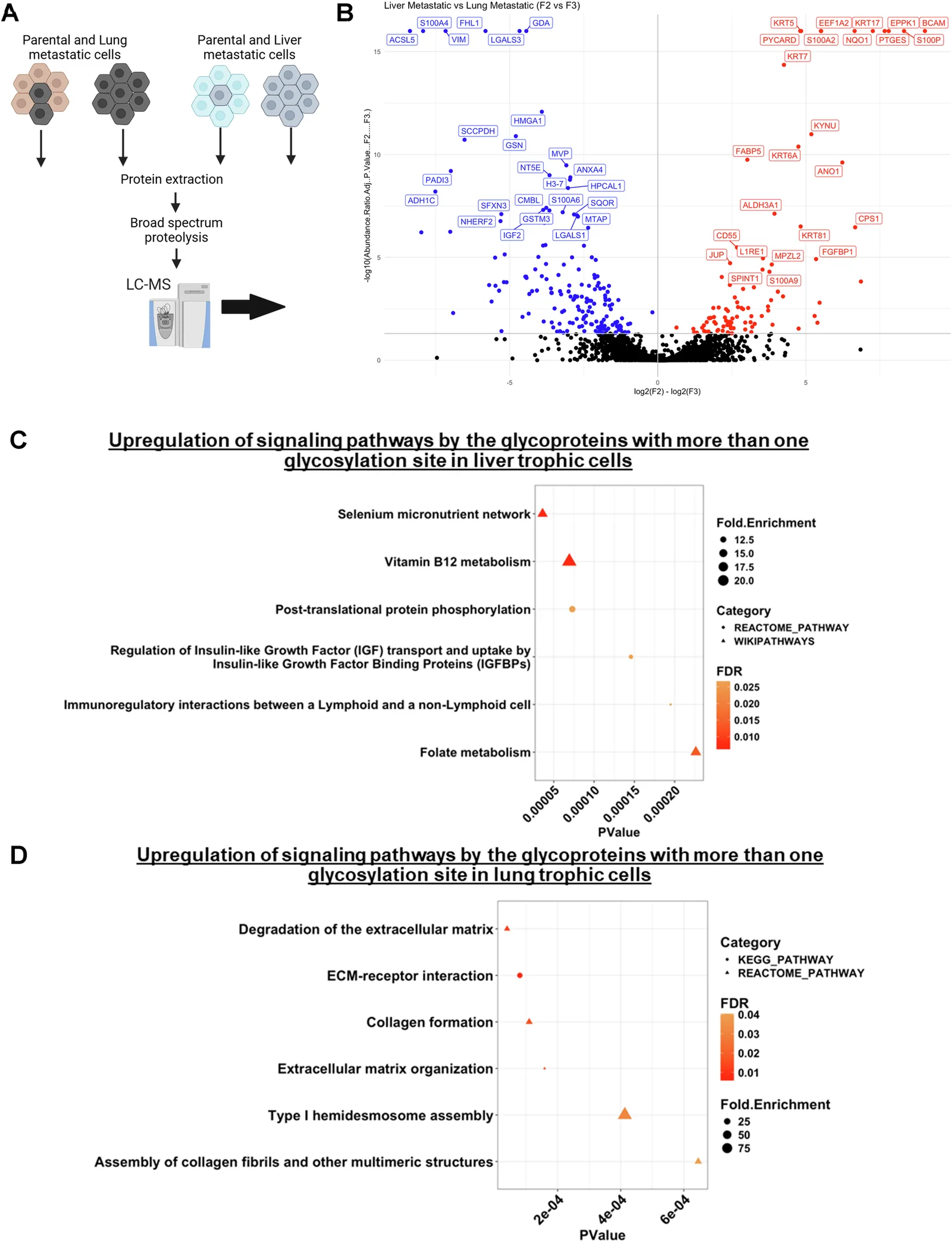
Proteomic profiling reveals glycoprotein-driven changes in cellular pathways and EMT signatures in organ-tropic PDAC cells. **A** Schematic representation of the proteomic workflow used to analyze glycoproteins, highlighting the identification and characterization of proteins with multiple glycosylation sites. **B** MS-based quantitative proteomic profiling of lung- and liver-tropic PDAC metastatic cell lines revealed distinct organ-specific protein expression signatures, reflecting differential adaptations to the respective metastatic microenvironments. **C**, **D** Pathway enrichment analysis of glycoproteomic data derived from mass spectrometry reveals distinct pathway activation patterns: pathways are enriched in liver-tropic PDAC cells (**C**), and signaling pathways are preferentially activated in lung-tropic PDAC cells (**D**), highlighting organ-specific adaptation mechanisms driven by differential glycosylation.

To define the proteomic basis underlying organ-specific metastatic behavior in PDAC, we performed comparative mass spectrometry-based proteomic analyses of liver- and lung-tropic metastatic PDAC cell lines along with their respective parental lines (Fig. 7A). Notably, these metastatic derivatives exhibit a reciprocal glycosyltransferase expression pattern, with liver-tropic cells enriched for GCNT3 and lung-tropic cells enriched for B3GNT3, providing the biological rationale for this comparative proteomic strategy. This approach revealed distinct protein expression landscapes between the two metastatic phenotypes, reinforcing the molecular divergence between liver- and lung-colonizing PDAC cells (Fig. 7B). Focusing on glycoproteins harboring one or more glycosylation sites, we conducted pathway enrichment analysis to identify cellular processes selectively active in each organ-tropic context. In liver-tropic cells, glycoproteins were enriched predominantly in pathways related to metabolic signaling, immune regulation, and cell survival (Fig. 7C). In contrast, lung-tropic cells were significantly enriched in extracellular matrix remodeling and structural reorganization pathways, which is consistent with the demands of pulmonary colonization (Fig. 7D). We also observed a striking difference in the expression of key epithelial‒mesenchymal transition (EMT) regulators. Vimentin (VIM) was downregulated in GCNT3-expressing liver-tropic cells, whereas basal cell adhesion molecule (BCAM) was upregulated, consistent with organ-specific epithelial–mesenchymal adaptations (Fig. 7B). In a matched parental comparison, Liver Met 1 cells exhibited significant downregulation of Vimentin (VIM) relative to COLO357 cells, indicating a liver microenvironment–driven epithelial shift rather than baseline cell line–specific differences (Supplementary Fig. 5J). Importantly, the loss of GTs altered the expression of these EMT markers. B3GNT3 KO in lung-tropic cells led to an increase in the expression of BCAM, whereas GCNT3 KO or talniflumate treatment in liver-tropic cells increased VIM expression, which was associated with epithelial adhesion and tissue-specific anchoring properties (Supplementary Fig. 5K). These reciprocal EMT signatures were validated in a second metastatic PDAC model, further supporting their relevance in organ-specific adaptation (Supplementary Fig. 5L). Collectively, these findings suggest that glycoprotein-mediated signaling not only dictates pathway activation but also reinforces EMT phenotypes aligned with organ-specific metastatic competency in PDAC.

## Discussion

Following the inception of the “seed and soil theory” of cancer metastasis, numerous investigations have endeavored to clarify the crucial cellular factors influencing organ-specific metastasis [32–36]. While prior studies have implicated glycosylation in tumor progression, most have not addressed how distinct GT programs are selectively engaged in a site-specific manner during metastatic colonization [37]. Our study identified GCNT3 and B3GNT3 as key regulators of metastatic colonization to the liver and lungs, respectively, and elucidated their functional roles. These findings advance our understanding of how GTs facilitate metastasis [38–40] and microenvironment-specific survival mechanisms [26, 41]. We observed distinct transcriptional profiles of GTs, with GCNT3 and B3GNT3 selectively enriched in liver- and lung-derived metastatic cells, respectively. Such transcriptional divergence in GT expression is consistent with emerging evidence that metastatic competence can be shaped by factors such as the stromal microenvironment [42], the Immune landscape [43], and vascular niches [44]. The observation that GCNT3 and B3GNT3 are critical elements of the glycoproteomic landscape in PDAC metastasis reinforces prior studies linking GTs to niche-specific colonization across various tumor types [14, 22].

Functionally, silencing B3GNT3 in lung-enriched metastatic cells led to marked reductions in clonogenicity, migration, and invasion in PDAC cells. The differential clonogenicity responses observed between liver- and lung-tropic CRISPR KO models likely reflect the pathway hierarchy of the targeted enzymes. The upstream loss of the core-branching GCNT3 produces a non-redundant structural dependency, while downstream B3GNT3-mediated glycan elongation is more functionally redundant and adaptable in the lung-metastatic context. Furthermore, the human mimicking metastatic model showed that B3GNT3 colonized the lung, which suggests the impact of this GT on metastatic survival. These observations are consistent with the prior studies, which show that the metastatic outgrowth depends on adaptive metabolic rewiring [45] and maintenance of stem-like traits [46] that are selectively reinforced by organ-specific microenvironments. Glycosylation has emerged as one such key regulator of this process by modulating receptor signaling [47], nutrient uptake [48], and cell–matrix interactions [37], by modulating GT expression. Interestingly, GCNT3 inhibition via both pharmacologic and genetic ablation reduced these same tumorigenic traits in liver-tropic PDAC cells and diminished metastatic colonization in decellularized liver scaffolds. These parallel findings suggest that GCNT3 and B3GNT3 confer organ-specific advantages, possibly by tuning glycan structures in a manner that supports their growth by modulating metabolism or even stemness within distinct metastatic niches [20].

Proteomic analysis revealed differential expressions of EMT-associated proteins, with GCNT3-expressing liver-metastatic cells exhibiting increased BCAM and decreased vimentin levels, whereas B3GNT3-expressing lung-metastatic cells exhibited the opposite pattern. This reciprocal regulation indicates a GT-EMT axis in PDAC metastasis, in which GTs may post-translationally modify adhesion molecules or EMT regulators, affecting their stability or receptor engagement. These findings align with the current literature, which shows that EMT is not a binary process but a spectrum of plastic states that are selectively stabilized by organ-specific microenvironments during metastatic colonization [49, 50]. Depletion of GCNT3 or B3GNT3 significantly reduced the metastatic burden to the respective organ, as confirmed by CK19 staining. These data establish a causal role for these GTs in site-specific metastatic colonization, which aligns with the concept that tumor-intrinsic glycosylation programs dictate niche adaptation [47, 51].

Our findings also resonate with broader paradigms in metastatic biology. Analogous to metabolic rewiring [20] or immune evasion [52], glycan remodeling is an adaptive mechanism that enables disseminated tumor cells to thrive in different microenvironments. The distinct sialylation patterns observed in B3GNT3-expressing lung metastases compared with the T-antigen accessibility retained in GCNT3-high liver metastases underscore how differential glycosylation may influence not only intrinsic cell behaviors but also interactions with stromal and immune components of the metastatic niche. The functional consequences of differential sialylation on immune recognition or stromal engagement were not directly interrogated, warranting future investigation into glycan-mediated niche interactions.

Although our study revealed a compelling link between GT expression and metastatic organotropism, several questions remain. For example, the upstream regulatory networks controlling GCNT3 and B3GNT3 expressions in PDAC remain undefined. Transcriptional control may involve niche-induced signaling cues or chromatin-level changes specific to metastatic organ sites. Additionally, although our focus was on GT expression in tumor cells, the contribution of stromal-derived glycan-modifying enzymes cannot be ruled out, especially in shaping the premetastatic niche. Because all in vivo experiments were performed in immune-deficient mouse models, future studies employing immune-competent or humanized systems will be necessary to define the immunologic contributions of glycosylation-dependent metastatic programs.

From a translational perspective, GCNT3 and B3GNT3 may serve as prognostic markers or therapeutic targets in metastatic PDAC. GCNT3 and B3GNT3 expressions and their associations with reduced patient survival, as observed in TCGA and clinical data analyses, highlight their potential clinical relevance. Inhibitors such as talniflumate, which target GCNT3, warrant further exploration to define clinically achievable concentrations through integrated pharmacokinetic and pharmacodynamic analyses, which will be essential for evaluating their translational and therapeutic potential. Similar efforts could be directed at identifying drug targets or studying the downstream glycan structures of B3GNT3. Moreover, our findings provide a rationale for the development of glycoproteomic biomarkers, enabling the stratification of PDAC patients based on metastatic risk and site-specific vulnerability. Future studies leveraging mouse models and clinically annotated PDAC cohorts with long-term follow-up and molecular subtyping will be essential to determine whether expression in primary tumors can predict organ-specific metastatic outcomes or stratify patients by PDAC subtype.

Overall, this study delineates how GTs such as GCNT3 and B3GNT3 orchestrate organ-specific metastasis in PDAC. By integrating glycosylation signatures with metastatic phenotypes, we reveal a new layer of regulation in site-selective colonization and propose that glycan remodeling is the fundamental driver of tumor adaptability in metastatic progression.

## Supplementary information


Supplementary Figure legends
Supplementary Figure 1
Supplementary Figure 2
Supplementary Figure 3
Supplementary Figure 4
Supplementary Figure 5
Supplementary file: Materials and Methods


## Data Availability

The datasets analyzed during the current study are available from the corresponding author on reasonable request.

## References

[1] Siegel RL, Giaquinto AN, Jemal A. Cancer statistics, 2024. CA Cancer J Clin. 2024;74:12–49. 38230766 10.3322/caac.21820

[2] Rahib L, Wehner MR, Matrisian LM, Nead KT. Estimated Projection of US Cancer Incidence and Death to 2040. JAMA Netw Open. 2021;4:e214708. 33825840 10.1001/jamanetworkopen.2021.4708 PMC8027914

[3] Collaborators GBDPC. The global, regional, and national burden of pancreatic cancer and its attributable risk factors in 195 countries and territories, 1990-2017: a systematic analysis for the Global Burden of Disease Study 2017. Lancet Gastroenterol Hepatol. 2019;4:934–47. 10.1016/S2468-1253(19)30347-4 PMC7026711 31648972

[4] Vincent A, Herman J, Schulick R, Hruban RH, Goggins M. Pancreatic cancer. Lancet. 2011;378:607–20. 21620466 10.1016/S0140-6736(10)62307-0 PMC3062508

[5] Liu Z, Chen J, Ren Y, Liu S, Ba Y, Zuo A, et al. Multi-stage mechanisms of tumor metastasis and therapeutic strategies. Signal Transduct Target Ther. 2024;9:270. 39389953 10.1038/s41392-024-01955-5 PMC11467208

[6] Conroy T, Hammel P, Hebbar M, Ben Abdelghani M, Wei AC, Raoul JL, et al. FOLFIRINOX or gemcitabine as adjuvant therapy for pancreatic cancer. N Engl J Med. 2018;379:2395–406. 30575490 10.1056/NEJMoa1809775

[7] Mayuko O, Tsunenari T, Einama T, Ichio K, Konno F, Kobayashi K, et al. Pancreatic cancer with liver metastasis maintaining complete response with gemcitabine monotherapy: a case report. Oncol Lett. 2024;28:370. 38933809 10.3892/ol.2024.14503 PMC11200152

[8] Li Q, Bai L, Xing J, Liu X, Liu D, Hu X. Risk assessment of liver metastasis in pancreatic cancer patients using multiple models based on machine learning: a large population-based study. Dis Markers. 2022;2022:1586074. 35634443 10.1155/2022/1586074 PMC9132665

[9] Decoster C, Gilabert M, Autret A, Turrini O, Oziel-Taieb S, Poizat F, et al. Heterogeneity of metastatic pancreatic adenocarcinoma: Lung metastasis show better prognosis than liver metastasis-a case control study. Oncotarget. 2016;7:45649–55. 27286454 10.18632/oncotarget.9861 PMC5216749

[10] Zhao P, Jiang Q, Xue K, Liu X, Tian B. The role of pulmonary metastasectomy in patients suffering pancreatic ductal adenocarcinoma with lung metastases: a systematic review and meta-analysis. Front Surg. 2025;12:1535212. 40084343 10.3389/fsurg.2025.1535212 PMC11903735

[11] Chatterjee D, Rashid A, Wang H, Katz MH, Wolff RA, Varadhachary GR, et al. Tumor invasion of muscular vessels predicts poor prognosis in patients with pancreatic ductal adenocarcinoma who have received neoadjuvant therapy and pancreaticoduodenectomy. Am J Surg Pathol. 2012;36:552–9. 22301496 10.1097/PAS.0b013e318240c1c0 PMC3310954

[12] Radhakrishnan P, Dabelsteen S, Madsen FB, Francavilla C, Kopp KL, Steentoft C, et al. Immature truncated O-glycophenotype of cancer directly induces oncogenic features. Proc Natl Acad Sci USA. 2014;111:E4066–4075. 25118277 10.1073/pnas.1406619111 PMC4191756

[13] Gupta R, Leon F, Rauth S, Batra SK, Ponnusamy MP. A systematic review on the implications of O-linked glycan branching and truncating enzymes on cancer progression and metastasis. Cells. 2020;9:446. 10.3390/cells9020446 PMC7072808 32075174

[14] Lumibao JC, Tremblay JR, Hsu J, Engle DD. Altered glycosylation in pancreatic cancer and beyond. J Exp Med. 2022;219:e20211505. 10.1084/jem.20211505 PMC9086500 35522218

[15] Rodriguez E, Boelaars K, Brown K, Madunic K, van Ee T, Dijk F, et al. Analysis of the glyco-code in pancreatic ductal adenocarcinoma identifies glycan-mediated immune regulatory circuits. Commun Biol. 2022;5:41. 35017635 10.1038/s42003-021-02934-0 PMC8752754

[16] Leon F, Seshacharyulu P, Nimmakayala RK, Chugh S, Karmakar S, Nallasamy P, et al. Reduction in O-glycome induces differentially glycosylated CD44 to promote stemness and metastasis in pancreatic cancer. Oncogene. 2022;41:57–71. 34675409 10.1038/s41388-021-02047-2 PMC8727507

[17] Zhang T, van Die I, Tefsen B, van Vliet SJ, Laan LC, Zhang J, et al. Differential O- and glycosphingolipid glycosylation in human pancreatic adenocarcinoma cells with opposite morphology and metastatic behavior. Front Oncol. 2020;10:732. 32582529 10.3389/fonc.2020.00732 PMC7280451

[18] Sagar S, Leiphrakpam PD, Thomas D, McAndrews KL, Caffrey TC, Swanson BJ, et al. MUC4 enhances gemcitabine resistance and malignant behaviour in pancreatic cancer cells expressing cancer-associated short O-glycans. Cancer Lett. 2021;503:91–102. 33485947 10.1016/j.canlet.2021.01.015 PMC7981252

[19] Hofmann BT, Schluter L, Lange P, Mercanoglu B, Ewald F, Folster A, et al. COSMC knockdown mediated aberrant O-glycosylation promotes oncogenic properties in pancreatic cancer. Mol Cancer. 2015;14:109. 26021314 10.1186/s12943-015-0386-1 PMC4447007

[20] Varadharaj V, Petersen W, Batra SK, Ponnusamy MP. Sugar symphony: glycosylation in cancer metabolism and stemness. Trends Cell Biol. 2024;35:412–425. 39462722 10.1016/j.tcb.2024.09.006 PMC12032065

[21] Marimuthu S, Lakshmanan I, Muniyan S, Gautam SK, Nimmakayala RK, Rauth S, et al. MUC16 promotes liver metastasis of pancreatic ductal adenocarcinoma by upregulating NRP2-associated cell adhesion. Mol Cancer Res. 2022;20:1208–21. 35533267 10.1158/1541-7786.MCR-21-0888 PMC9635595

[22] Gupta R, Leon F, Thompson CM, Nimmakayala R, Karmakar S, Nallasamy P, et al. Global analysis of human glycosyltransferases reveals novel targets for pancreatic cancer pathogenesis. Br J Cancer. 2020;122:1661–72. 32203219 10.1038/s41416-020-0772-3 PMC7251111

[23] Marimuthu S, Batra SK, Ponnusamy MP. Pan-cancer analysis of altered glycosyltransferases confers poor clinical outcomes. Clin Transl Discov. 2022;2:e100. 10.1002/ctd2.100 PMC9302706 35875597

[24] Kitamura N, Iwamura T, Taniguchi S, Yamanari H, Kawano MA, Hollingsworth K, et al. High collagenolytic activity in spontaneously highly metastatic variants derived from a human pancreatic cancer cell line (SUIT-2) in nude mice. Clin Exp Metastasis. 2000;18:561–71. 11688961 10.1023/a:1011900818419

[25] Bruns CJ, Harbison MT, Kuniyasu H, Eue I, Fidler IJ. In vivo selection and characterization of metastatic variants from human pancreatic adenocarcinoma by using orthotopic implantation in nude mice. Neoplasia. 1999;1:50–62. 10935470 10.1038/sj.neo.7900005 PMC1764837

[26] Bindeman WE, Fingleton B. Glycosylation as a regulator of site-specific metastasis. Cancer Metastasis Rev. 2022;41:107–29. 34967926 10.1007/s10555-021-10015-1 PMC8930623

[27] Murugaesu N, Iravani M, van Weverwijk A, Ivetic A, Johnson DA, Antonopoulos A, et al. An in vivo functional screen identifies ST6GalNAc2 sialyltransferase as a breast cancer metastasis suppressor. Cancer Discov. 2014;4:304–17. 24520024 10.1158/2159-8290.CD-13-0287

[28] Zhang Y, Sun L, Lei C, Li W, Han J, Zhang J. et al. A sweet warning: mucin-type O-glycans in cancer. Cells. 2022;11:3666. 10.3390/cells11223666 PMC9688771 36429094

[29] Mereiter S, Balmana M, Campos D, Gomes J, Reis CA. Glycosylation in the era of cancer-targeted therapy: where are we heading? Cancer Cell. 2019;36:6–16. 31287993 10.1016/j.ccell.2019.06.006

[30] Davies H, Bignell GR, Cox C, Stephens P, Edkins S, Clegg S, et al. Mutations of the BRAF gene in human cancer. Nature. 2002;417:949–54. 12068308 10.1038/nature00766

[31] Rao CV, Janakiram NB, Madka V, Kumar G, Scott EJ, Pathuri G, et al. Small-molecule inhibition of GCNT3 disrupts mucin biosynthesis and malignant cellular behaviors in pancreatic cancer. Cancer Res. 2016;76:1965–74. 26880801 10.1158/0008-5472.CAN-15-2820

[32] Hoshino A, Costa-Silva B, Shen TL, Rodrigues G, Hashimoto A, Tesic Mark M, et al. Tumour exosome integrins determine organotropic metastasis. Nature. 2015;527:329–35. 26524530 10.1038/nature15756 PMC4788391

[33] Fares J, Fares MY, Khachfe HH, Salhab HA, Fares Y. Molecular principles of metastasis: a hallmark of cancer revisited. Signal Transduct Target Ther. 2020;5:28. 32296047 10.1038/s41392-020-0134-x PMC7067809

[34] Lusby R, Demirdizen E, Inayatullah M, Kundu P, Maiques O, Zhang Z, et al. Pan-cancer drivers of metastasis. Mol Cancer. 2025;24:2. 39748426 10.1186/s12943-024-02182-w PMC11697158

[35] Agrawal A, Javanmardi Y, Watson SA, Serwinski B, Djordjevic B, Li W, et al. Mechanical signatures in cancer metastasis. NPJ Biol Phys Mech. 2025;2:3. 39917412 10.1038/s44341-024-00007-x PMC11794153

[36] Alard A, Katsara O, Rios-Fuller T, Parra C, Ozerdem U, Ernlund A, et al. Breast cancer cell mesenchymal transition and metastasis directed by DAP5/eIF3d-mediated selective mRNA translation. Cell Rep. 2023;42:112646. 37314929 10.1016/j.celrep.2023.112646 PMC10895648

[37] Oliveira-Ferrer L, Legler K, Milde-Langosch K. Role of protein glycosylation in cancer metastasis. Semin Cancer Biol. 2017;44:141–52. 28315783 10.1016/j.semcancer.2017.03.002

[38] Hung CH, Wu SY, Yao CD, Yeh HH, Lin CC, Chu CY, et al. Defective N-glycosylation of IL6 induces metastasis and tyrosine kinase inhibitor resistance in lung cancer. Nat Commun. 2024;15:7885. 39251588 10.1038/s41467-024-51831-7 PMC11385228

[39] Li X, Wang X, Tan Z, Chen S, Guan F. Role of glycans in cancer cells undergoing epithelial-mesenchymal transition. Front Oncol. 2016;6:33. 26925388 10.3389/fonc.2016.00033 PMC4756103

[40] Ponnusamy MP, Seshacharyulu P, Lakshmanan I, Vaz AP, Chugh S, Batra SK. Emerging role of mucins in epithelial to mesenchymal transition. Curr Cancer Drug Targets. 2013;13:945–56. 24168188 10.2174/15680096113136660100 PMC3924542

[41] Biray Avci C, Goker Bagca B, Nikanfar M, Takanlou LS, Takanlou MS, Nourazarian A. Tumor microenvironment and cancer metastasis: molecular mechanisms and therapeutic implications. Front Pharm. 2024;15:1442888. 10.3389/fphar.2024.1442888 PMC11588459 39600368

[42] Ligorio M, Sil S, Malagon-Lopez J, Nieman LT, Misale S, Di Pilato M, et al. Stromal microenvironment shapes the intratumoral architecture of pancreatic cancer. Cell. 2019;178:160–175.e127. 31155233 10.1016/j.cell.2019.05.012 PMC6697165

[43] Kitamura T, Qian BZ, Pollard JW. Immune cell promotion of metastasis. Nat Rev Immunol. 2015;15:73–86. 25614318 10.1038/nri3789 PMC4470277

[44] Augustin HG, Koh GY. Organotypic vasculature: from descriptive heterogeneity to functional pathophysiology. Science. 2017;357:eaal2379. 10.1126/science.aal2379 28775214

[45] Pavlova NN, Zhu J, Thompson CB. The hallmarks of cancer metabolism: Still emerging. Cell Metab. 2022;34:355–77. 35123658 10.1016/j.cmet.2022.01.007 PMC8891094

[46] Malanchi I, Santamaria-Martinez A, Susanto E, Peng H, Lehr HA, Delaloye JF, et al. Interactions between cancer stem cells and their niche govern metastatic colonization. Nature. 2011;481:85–89. 22158103 10.1038/nature10694

[47] Pinho SS, Reis CA. Glycosylation in cancer: mechanisms and clinical implications. Nat Rev Cancer. 2015;15:540–55. 26289314 10.1038/nrc3982

[48] Dennis JW, Brewer CF. Density-dependent lectin-glycan interactions as a paradigm for conditional regulation by posttranslational modifications. Mol Cell Proteom. 2013;12:913–20. 10.1074/mcp.R112.026989 PMC3617338 23378517

[49] Pastushenko I, Blanpain C. EMT transition states during tumor progression and metastasis. Trends Cell Biol. 2019;29:212–26. 30594349 10.1016/j.tcb.2018.12.001

[50] Brabletz T, Kalluri R, Nieto MA, Weinberg RA. EMT in cancer. Nat Rev Cancer. 2018;18:128–34. 29326430 10.1038/nrc.2017.118

[51] Reticker-Flynn NE, Bhatia SN. Aberrant glycosylation promotes lung cancer metastasis through adhesion to galectins in the metastatic niche. Cancer Discov. 2015;5:168–81. 25421439 10.1158/2159-8290.CD-13-0760 PMC4367955

[52] Zhu Q, Wang H, Chai S, Xu L, Lin B, Yi W, et al. O-GlcNAcylation promotes tumor immune evasion by inhibiting PD-L1 lysosomal degradation. Proc Natl Acad Sci USA. 2023;120:e2216796120. 36943877 10.1073/pnas.2216796120 PMC10068856

