# Supplementary material for: Distinct glycosyltransferase expression governs organ-specific survival in pancreatic cancer metastasis
Source: Oncogene. 2026 Jul 30;45(36):3858–73. doi: 10.1038/s41388-026-03896-5 (PMC13529584; doi:10.1038/s41388-026-03896-5)
Supplement: Supplementary file 1 [file 41388_2026_3896_MOESM1_ESM.docx]

**SUPPLEMENTARY FIGURE LEGENDS**

**Supplementary Figure 1. Organ-specific glycosyltransferase expression in PDAC models.**

**A**, Heatmap of global glycosyltransferase (GT) gene expression profiles obtained from transcriptional analysis of lung-tropic (Lung Met1) and liver-tropic (Liver Met1) PDAC metastatic cell lines compared with their respective parental PDAC cell lines.

**B and C**, ddPCR quantification of GCNT3 (**B**) and B3GNT3 (**C**) transcript levels in the first metastatic PDAC model (Liver Met1 and Lung Met1). The expression levels were normalized to those of β-actin.

**D and E**, ddPCR quantification of GCNT3 (**D**) transcript levels in an independent second metastatic PDAC model (Liver Met2 and Lung Met2). The expression levels were normalized to those of GAPDH (**E**).

**F and G**, ddPCR quantification of B3GNT3 (**F**) transcript levels in an independent second metastatic PDAC model (Liver Met2 and Lung Met2). The expression levels were normalized to those of GAPDH (**G**).

**H**, Ponceau S staining of the membrane showing total protein levels and confirming equal loading across samples.

**I and J,** Absolute quantification of ST8SIA6 expression by droplet digital PCR (ddPCR) in liver- and lung-tropic metastatic cell lines. The expression levels were normalized to those of β-actin.

**K,** Analysis of ST8SIA6 expression in metastatic tissues derived from the KPC (Kras^G12D; Trp53^R172H; Pdx1-Cre) mouse model, demonstrating differential expression between lung and liver metastases consistent with organ-specific patterns.

**L,** Kaplan–Meier analysis of overall survival stratified by high versus low ST8SIA6 expression in patient cohorts.

**M,** Kaplan–Meier analysis of disease-free survival based on ST8SIA6 expression levels, showing no significant association with clinical outcome.

The data are presented as the means ± SEMs. *P < 0.05; **P < 0.01; ***P < 0.001; NS, not significant.

**Supplementary Figure 2. Patient-level spatial and primary tumor expression analysis of GCNT3 and B3GNT3**

**A,** Spatial transcriptomic analysis of matched primary tumor samples to the liver metastatic sample in Figure 3H from dataset GSE274557, showing the distribution of GCNT3 and B3GNT3 expression within primary PDAC tissue.

**(B-F)** Patient-level analysis demonstrating differential expression patterns of GCNT3 and B3GNT3 across individual primary treatment naive patient tumor samples, highlighting inter-patient variability and gene-specific expression trends.

**(G)** Clinical annotation of the patient cohort used for spatial transcriptomic analysis, including corresponding metastatic organ involvement (liver or lung).

**Supplementary Figure 3. Prognostic and tumor purity associations of GCNT3 and B3GNT3.**

**A-C**, Kaplan–Meier survival analysis demonstrated that high expression of GCNT3 (**A**) B3GNT3 (**B**) and both (**C**) is significantly associated with reduced overall survival in PDAC patients.

**D-F**, High expression of GCNT3 (**D**) B3GNT3 (**E**) and both (**F**)was also correlated with significantly reduced disease-free survival.

**G and H**, Analysis of ABSOLUTE purity scores in TCGA PDAC datasets revealed a strong positive correlation between tumor cell content and GCNT3 (**G**) and B3GNT3 (**H**) expression levels.

**I**, Compared with those in normal pancreas, both GCNT3 and B3GNT3 rank among the top differentially expressed glycogenes in PDAC, as determined from integrated transcriptomic datasets, highlighting their potential roles in glycosylation-driven tumor progression and metastatic adaptation.

**J,** Analysis of GCNT3 expression in the GSE71729 PDAC cohort stratified by molecular subtype, showing significantly higher expression in classical tumors compared with basal-like tumors.

**K,** Analysis of B3GNT3 expression across classical and basal-like PDAC subtypes in GSE71729, demonstrating no significant subtype-specific difference.

**L,** Immunoblot analysis of EMT markers in metastatic PDAC cell models showing increased E-cadherin expression in liver-tropic cells and elevated ZEB1 expression in lung-tropic cells, consistent with epithelial and basal-like/mesenchymal phenotypes, respectively.

**Supplementary Figure 4. Functional consequences of GT inhibition in in vitro and ex vivo models.**

**A**, Western blot confirming CRISPR/Cas9-mediated knockout (KO) of B3GNT3 in an independent lung-tropic PDAC model (second metastatic model).

**B and C**, B3GNT3 KO clones exhibited significantly reduced colony formation compared with the corresponding control clones (**B**), validating the impact of B3GNT3 loss on tumorigenic potential across independent models and quantification (**C**).

**D and E**, Transwell migration assays confirmed the consistent impairment of cell migration following B3GNT3 KO (**D**) in the second lung-tropic PDAC model, and the results were quantified (**E**).

**F**, Wound-healing assays revealed diminished wound closure in B3GNT3 KO clones than in control clones, which was consistent with impaired motility.

**G**, *Ex vivo* lung metastasis model using decellularized lung scaffolds confirmed impaired 3D spheroid survival in B3GNT3 KO lung-tropic PDAC cells, functionally validating the role of B3GNT3 in supporting lung-specific metastatic colonization.

**H-J,** Representative images of liver- and lung-tropic metastatic spheroids cultured within liver, lung microenvironment-mimetic conditions and also with O glycan inhibitor (BAG), showing preferential survival of each metastatic population within its corresponding organ niche.

**Supplementary Figure 5. Functional and molecular validation of organ-tropic PDAC and associated epithelial–mesenchymal remodeling**

**A**, IC50 curve demonstrating a dose-dependent reduction in GCNT3 protein expression following talniflumate treatment in liver-tropic PDAC cells, confirming effective pharmacologic inhibition of GCNT3 glycosyltransferase activity.

**B**, Western blotting confirmed the successful CRISPR/Cas9-mediated knockout (KO) of GCNT3 in liver-tropic PDAC cells, validating the genetic ablation model used for subsequent functional assays.

**C and D**, Clonogenic assays show that GCNT3 KO liver-tropic PDAC cells form significantly fewer colonies than isogenic control cells do (**I**), mirroring the results observed with pharmacological inhibition and quantification (**J**).

**E and F**, Transwell migration assays confirmed that GCNT3 KO cells exhibit markedly reduced motility relative to control cells (**K**), which is consistent with a critical role for GCNT3 in facilitating PDAC cell migration and quantification (**L**).

**G**, Wound-healing assays further demonstrated impaired wound closure in GCNT3 KO clones compared with controls, corroborating the loss of migratory capacity upon genetic depletion of GCNT3.

**H,** Representative histological images showing absence of detectable lung metastasis following implantation of liver-tropic metastatic cells.

**I,** Histological analysis demonstrating minimal liver colonization by lung-tropic metastatic cells, observed as small micro-metastatic foci.

**J,** Mass spectrometry–based proteomic comparison between Liver Met 1 cells and their matched parental COLO357 cells showing significant downregulation of Vimentin (VIM) in Liver Met 1 cells, consistent with liver microenvironment–associated epithelial adaptation rather than baseline cell line differences.

**K,** Western blot analysis validated the proteomic findings by confirming the reciprocal expression of epithelial‒mesenchymal transition (EMT)-associated markers: vimentin (VIM), a mesenchymal marker enriched in lung-tropic PDAC cells, and basal cell adhesion molecule (BCAM), an epithelial marker selectively enriched in liver-tropic PDAC cells, which are correlated with their distinct invasive phenotypes. The expression pattern changes on the basis of the availability of GTs, B3GNT3 and GCNT3.

**L**, Western blot analysis of EMT markers validated the reciprocal expression of vimentin (VIM) and basal cell adhesion molecule (BCAM) in a second metastatic PDAC model, confirming the EMT-associated glycoprotein remodeling observed in the primary models.
