# Supplementary material for: Distinct glycosyltransferase expression governs organ-specific survival in pancreatic cancer metastasis
Source: Oncogene. 2026 Jul 30;45(36):3858–73. doi: 10.1038/s41388-026-03896-5 (PMC13529584; doi:10.1038/s41388-026-03896-5)
Supplement: Supplementary file 2 [file 41388_2026_3896_MOESM7_ESM.docx]

**MATERIALS AND METHODS**

**Generation of stable GCNT3- and B3GNT3-KD cell lines:** Stable KD of GCNT3 and B3GNT3 was achieved in the first metastatic model (Liver met 1 and Lung met 1)via shRNA-mediated gene silencing. Control cells were transduced with a nontargeting scrambled (SCR) shRNA construct. The specific shRNA sequences used are listed in Table S5. To produce lentiviral particles, HEK293T cells were cotransfected with the shRNA plasmids and packaging vectors via Lipofectamine 2000. Viral supernatants were harvested at 48- and 72-hours post-transfection and used to transduce target cells in the presence of polybrene (8 µg/mL). Forty-eight hours after infection, the transduced cells were selected by fluorescence-activated cell sorting (FACS) on the basis of GFP expression, yielding stable GCNT3- or B3GNT3-KD lines for downstream analysis.

**CRISPR/Cas9-mediated knockout of GCNT3 and B3GNT3**

CRISPR/Cas9-mediated knockout of GCNT3 or B3GNT3 was performed using pSpCas9(BB)-2A-GFP (PX458), which co-expresses SpCas9 and sgRNA under a U6 promoter and enables enrichment of transfected cells via GFP. Cells were seeded to achieve ~60–80% confluency at the time of transfection. PX458 plasmids encoding sgRNAs targeting GCNT3 or B3GNT3 were transfected using a Lipofectamine 3000 (Thermo Fisher Scientific) according to the manufacturer’s protocol. Briefly, plasmid DNA was diluted in serum-free medium and combined with the transfection reagent (and enhancer reagent, if applicable) to form DNA–lipid complexes, which were then added dropwise to cells maintained in complete growth medium. Medium was replaced the next day as needed, and cells were cultured for 48–72 h to allow Cas9/sgRNA expression. To enrich edited cells, GFP-positive cells were isolated by fluorescence-activated cell sorting (FACS) and either collected as a bulk population to generate clonal lines. Clones were expanded under standard culture conditions, and candidate KO clones were screened by western blotting for loss of GCNT3 or B3GNT3 protein relative to control cells. Verified KO clones were used for downstream experiments.

**Immunohistochemistry and Immunofluorescence:** Formalin-fixed paraffin-embedded tissue slides were incubated overnight at 58°C, cooled to room temperature, deparaffinized with 100% xylene (4 × 10 min), and rehydrated through graded ethanol solutions for 10 minutes each. For immunohistochemistry (IHC), endogenous peroxidase activity was quenched via the addition of 3% H₂O₂ in methanol for 1 hour in the dark, followed by antigen retrieval in 0.01 M citrate buffer (pH 6.8) at room temperature for 30 minutes. The slides were blocked with 2.5% horse serum for 1 hour, incubated overnight at 4°C with primary antibodies against GCNT3 and B3GNT3, and washed with PBS-T (3 × 10 min) and PBS (1 × 10 min). The following day, the slides were incubated with HRP-conjugated universal anti-mouse/rabbit IgG for 1 hour, developed using DAB substrate (Vector Labs), counterstained with hematoxylin, dehydrated through increasing alcohol concentrations, cleared in xylene (4 × 10 min), and mounted with Permount (Fisher Scientific). Pathological scoring was conducted by a board-certified pathologist via an intensity scale (0–3) and the percentage of positively stained cells (1: 0–24%, 2: 25–49%, 3: 50–74%, 4: 75–100%) in a blinded manner. For immunofluorescence (IF), after rehydration and rinsing with 15 minutes of tap water, antigen retrieval was performed similarly, and the tissue sections were blocked with 10% normal goat serum for 1 hour. Primary antibodies (GCNT3, B3GNT3) were applied overnight at 4°C, followed by washes with PBS-T or PBS and incubation with fluorophore-conjugated secondary antibodies for 1 hour. The slides were then washed in PBS and mounted with DAPI-containing medium for confocal microscopy.

**RNA isolation and quantitative PCR (qPCR)**: Total RNA was isolated and prepared from cells via the Qiagen RNeasy Kit (Qiagen, Valencia, CA, USA) according to the manufacturer’s instructions. cDNA synthesis reactions were performed via iScript reverse transcription reagents (Bio-Rad). qPCR was performed with 2 μL of cDNA, 10 μL of LightCycler 480 SYBR Green I master mix (Roche), and 5 μM of each primer. mRNA expression and fold change values are reported following the ΔΔCT method. The expression of all genes was normalized to that of internal controls and expressed relative to the indicated reference sample.

**LC‒MS/MS**: Peptide samples (approximately 1 μg) were first loaded onto a precolumn (Acclaim PepMap™ 100, 75 μm × 2 cm, nano Viper, Thermo Scientific) and subsequently separated on an analytical column (Acclaim PepMap™ RSLC, 75 μm × 50 cm, nano Viper, Thermo Scientific). Elution was carried out via the introduction of a 125-minute linear gradient of acetonitrile (ACN) ranging from 9% to 45% in 0.1% formic acid (FA) into the mass spectrometer via a nanospray ion source. The instrument settings included the following: a nanospray needle voltage of 1950 V in positive mode, column flow rate of 0.300 μL/min, loading pump flow rate of 4 μL/min, injection mode set to μL PickUp, MS1 scans acquired in the Orbitrap at a resolution of 120,000 with a scan range of 197–1500 m/z, and MS2 scans performed at a resolution of 15,000. The acquired mass spectra were searched against the Swiss-Prot database (*Homo sapiens* subset, 2019_06 release, 20,432 entries) via MASCOT software (version 2.6.2). The search parameters included enzyme specificity for trypsin with up to two missed cleavages, a precursor ion tolerance of ± 0.6 Da, and peptide charge states of +1, +2, and +3. Fixed modifications were set to carbamidomethylation on cysteine residues, and variable modifications included oxidation on methionine. Peptide and protein identifications were further validated via Scaffold software (version 4.8.7), with thresholds of 99% for protein identification, 90% for peptide identification, and a minimum of two unique peptides per protein.

**Immunoblot:** The cells were lysed in RIPA buffer (Thermo Fisher Scientific) supplemented with protease and phosphatase inhibitors (Roche). The lysates were then centrifuged at 13,000 × g for 25 minutes at 4°C, and the supernatants were collected. Protein concentrations were determined via the Lowry assay. Equal amounts of protein (20 µg per sample) were resolved on a 10% SDS‒PAGE gel and transferred to a polyvinylidene difluoride (PVDF) membrane (Millipore) via a semidry transfer system (Bio‒Rad). The membranes were blocked for 1 hour at room temperature in 5% nonfat dry milk prepared in PBST (phosphate-buffered saline with 0.1% Tween-20). After blocking, the membranes were incubated overnight at 4°C with primary antibodies diluted in PBST. Following primary antibody incubation, the membranes were washed three times for 10 minutes each in PBST. The membranes were then incubated with horseradish peroxidase (HRP)-conjugated secondary antibodies (Invitrogen) diluted in 3% nonfat dry milk in PBST for 1 hour at room temperature. After secondary antibody incubation, the membranes were washed three times for 10 minutes each in PBST. The protein bands were visualized via an enhanced chemiluminescence substrate (Thermo Fisher) and imaged via iBright. Protein expression was normalized to that of the corresponding loading control.

**PNA lectin Assay:** Cell-surface glycoproteins were enriched using the Pierce Cell Surface Protein Biotinylation and Isolation Kit (Thermo Fisher Scientific; Cat. No. A44390) following the manufacturer’s instructions, with downstream PNA lectin Western blotting to assess glycosylation changes. Cells were washed with ice-cold PBS and incubated with the kit-provided EZ-Link Sulfo-NHS-SS-Biotin (thiol-cleavable, amine-reactive) to label accessible primary amines on extracellular domains of cell-surface proteins. Suspension cells were gently pelleted, resuspended in PBS, and labeled similarly. After labeling, cells were processed according to the kit workflow and lysed in the supplied Lysis Buffer supplemented with protease and phosphatase inhibitor cocktails. Lysates were incubated on ice with intermittent mixing and clarified by centrifugation to remove insoluble material. Equal amounts of protein from each condition were applied to NeutrAvidin Agarose spin columns and incubated with end-over-end mixing at room temperature to capture biotinylated (cell-surface) proteins. Columns were washed repeatedly with the kit Wash Buffer to minimize non-specific binding. Captured proteins were eluted using the kit elution conditions containing 10 mM dithiothreitol (DTT) to reduce the disulfide bond within the biotin linker (Sulfo-NHS-SS-Biotin), thereby releasing purified surface proteins. Eluted cell-surface protein fractions were prepared in SDS sample buffer, resolved by SDS–PAGE, and transferred to PVDF membranes. Membranes were blocked and incubated with peanut agglutinin (PNA) lectin to detect PNA-reactive glycans on enriched cell-surface proteins. Membranes were stained with Ponceau S to verify equal protein loading. PNA lectin binding was quantified by measuring band intensity across three molecular weight regions (~25, ~50, and ~75 kDa). For each region, PNA signal intensity was quantified across all samples. The resulting values were normalized to total protein loading as assessed by Ponceau S membrane staining, and normalized intensities were used for graphical representation and statistical analysis.

**Colony Formation:** The clonogenic potential of the cells was assessed via a colony formation assay. The cells were trypsinized, counted, and seeded into 6-well plates at a density of 500 cells per well. The cells were allowed to grow in a humidified incubator at 37°C with 5% CO₂ for 10–14 days or until visible colonies formed. After the incubation period, the media was carefully aspirated, and the cells were gently washed with PBS. The colonies were then fixed with 4% paraformaldehyde for 15 minutes at room temperature. Following fixation, the cells were washed with PBS and stained with 0.5% crystal violet solution in 25% methanol for 30 minutes. The plates were washed thoroughly with tap water to remove excess stains and allowed to air dry. Colonies consisting of ≥50 cells were counted via ImageJ software.

**Transwell migration:** Cell migration was assessed via a transwell migration assay. The assay was performed using 24-well transwell chambers with 8.0 µm pore size polycarbonate membrane inserts (Corning). The cells were serum starved for 12–24 hours prior to the assay. After starvation, 2 × 10⁴ cells in 200 µL of serum-free medium were seeded into the upper chamber of the transwell insert. The lower chamber was filled with 600 µL of medium containing 10% fetal bovine serum (FBS) as a chemoattractant. The cells were allowed to migrate for 24 hours in a humidified incubator at 37°C with 5% CO₂. After the incubation period, nonmigrated cells on the upper surface of the membrane were carefully removed via a cotton swab. The migrated cells on the lower surface of the membrane were fixed with 4% paraformaldehyde for 15 minutes and then stained with 0.5% crystal violet for 30 minutes. The membranes were washed with PBS to remove excess stain, and the number of migrated cells was quantified by counting the cells in five random fields per membrane via ImageJ. The results are expressed as the average number of migrated cells per field.

**Wound Healing Assay:** Cells were seeded at a density of 5,000 cells per well into 96-well ImageLock plates (Essen BioScience) and allowed to adhere and reach confluence for 24 hours. A uniform scratch was then created using an Incucyte® WoundMaker (Sartorius) according to the manufacturer’s protocol. Following the scratch, the wells were gently washed with PBS to remove detached cells, and fresh media was added. The plates were transferred to the IncuCyte Live-Cell Analysis System, where images were acquired every hour for up to 48 hours. The relative wound density was quantified via the IncuCyte® Scratch Wound Analysis Software, which calculates the relative wound density over time.

**Spatial data analysis**: Publicly available spatial transcriptomics data were obtained from the Gene Expression Omnibus (GEO) under accession number GSE274557. Raw spatial gene expression matrices and associated metadata were downloaded and processed in R using the Seurat framework following established spatial analysis workflows. Dimensionality reduction was performed using principal component analysis, and spatial features were visualized using Seurat’s spatial plotting functions. Tissue regions were annotated using the provided metadata to distinguish primary pancreatic tumors, liver metastases, and lung metastases. Expression of GCNT3 and B3GNT3 was examined and compared across primary and metastatic tissues using spatial feature plots and quantitative expression summaries.

**Statistical Analysis**: All the statistical analyses were performed using GraphPad Prism (Version 10.0.2). Data are presented as mean ± S.E.M., unless otherwise stated. For experiments with n < 5, individual data points were plotted. Comparisons between two groups were performed using a two-tailed unpaired Student’s t-test for normally distributed data or Mann–Whitney U test for nonparametric distributions. Comparisons involving more than two groups were analyzed using one-way ANOVA followed by Tukey’s multiple-comparison test. P values < 0.05 were considered statistically significant. The number of biological and technical replicates for each experiment is indicated in the figure legends.
